# Reproducibility of graph measures at the subject level using resting‐state fMRI

**DOI:** 10.1002/brb3.1705

**Published:** 2020-07-02

**Authors:** Qian Ran, Tarik Jamoulle, Jolien Schaeverbeke, Karen Meersmans, Rik Vandenberghe, Patrick Dupont

**Affiliations:** ^1^ Laboratory for Cognitive Neurology Department of Neurosciences, KU Leuven Leuven Belgium; ^2^ Department of Radiology Xinqiao Hospital Chongqing China; ^3^ Alzheimer Research Centre KU Leuven Leuven Brain Instititute, KU Leuven Leuven Belgium; ^4^ Neurology Department University Hospitals Leuven (UZ Leuven) Leuven Belgium

**Keywords:** denoising, graph measures, network construction, reproducibility, resting‐state fMRI, test–retest variability

## Abstract

**Introduction:**

Graph metrics have been proposed as potential biomarkers for diagnosis in clinical work. However, before it can be applied in a clinical setting, their reproducibility should be evaluated.

**Methods:**

This study systematically investigated the effect of two denoising pipelines and different whole‐brain network constructions on reproducibility of subject‐specific graph measures. We used the multi‐session fMRI dataset from the Brain Genomics Superstruct Project consisting of 69 healthy young adults.

**Results:**

In binary networks, the test–retest variability for global measures was large at low density irrespective of the denoising strategy or the type of correlation. Weighted networks showed very low test–retest values (and thus a good reproducibility) for global graph measures irrespective of the strategy used. Comparing the test–retest values for different strategies, there were significant main effects of the type of correlation (Pearson correlation vs. partial correlation), the (partial) correlation value (absolute vs. positive vs. negative), and weight calculation (based on the raw (partial) correlation values vs. based on transformed *Z*‐values). There was also a significant interaction effect between type of correlation and weight calculation. Similarly as for the binary networks, there was no main effect of the denoising pipeline.

**Conclusion:**

Our results demonstrated that normalized global graph measures based on a weighted network using the absolute (partial) correlation as weight were reproducible. The denoising pipeline and the granularity of the whole‐brain parcellation used to define the nodes were not critical for the reproducibility of normalized graph measures.

## Graphical abstract

We used the multi‐session fMRI dataset from the Brain Genomics Superstruct Project consisting of 69 healthy young adults. Our results demonstrated that normalized global graph measures based on a weighted network using the absolute (partial) correlation as weight were reproducible. The denoising pipeline and the granularity of the whole‐brain parcellation used to define the nodes were not critical for the reproducibility of normalized graph measures.

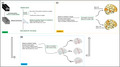

## 1 INTRODUCTION

Resting‐state fMRI (rs‐fMRI) is a task‐free and an easy‐to‐use tool for neuroscientific data acquisition. It is used to detect spontaneous low frequency (<0.1 Hz) fluctuations of the brain by blood‐oxygen‐level‐dependent (BOLD) signals during a state of rest (Biswal, Yetkin, Haughton, & Hyde, 1995; Smith et al., 2013). Those fluctuations are highly organized across discrete brain regions (Greicius, Krasnow, Reiss, & Menon, 2003). Functional connectivity analysis is a way to analyze how distant brain regions interact and graph theory can be used to quantify performance of the entire network (Bullmore & Sporns, 2009; Park & Friston, 2013; Sporns, 2011).

Many studies have reported that alterations of interactions among distant brain regions and dysfunction of networks are closely related to brain diseases, such as epilepsy (Burianová et al., 2017), Alzheimer's disease (Greicius, Srivastava, Reiss, & Menon, 2004; Hallquist & Hillary, 2018; Johnson, Sperling, & Sepulcre, 2013) and among others. Petrella (2011) proposed the use of graph metrics as potential biomarkers and diagnostic tools in clinical work. However, before graph measures can be widely applied as a biomarker in a clinical setting, it is critical that a number of properties of graph measures should be evaluated rigorously. These properties include simplicity, robustness, and test–retest variability.

The reproducibility of rs‐fMRI based graph measures faces a big challenge, namely how to effectively handle BOLD signals contaminated by noise (Bianciardi et al., 2009) and how to reduce the influence of noise on the reproducibility of graph measures. The main causes of this contamination are head motion and non‐neuronal physiological fluctuations. Head motion, even very subtle movement, has been demonstrated to have a negative impact on BOLD signals (Parkes, Fulcher, Yücel, & Fornito, 2018; Satterthwaite et al., 2012, 2019). Therefore, the influence caused by head motion and non‐neuronal physiological fluctuations should be removed as much as possible to reduce the impact on functional connectivity.

Research in denoising techniques reducing the influence of noise in BOLD signals attracted quite some attention (Satterthwaite et al., 2019). Denoising techniques can have an impact on the reproducibility of graph measures. The most common strategies for denoising typically correct the signal using three types of information: (a) head movement parameters (Friston, Williams, Howard, Frackowiak, & Turner, 1996; Satterthwaite et al., 2013); (b) physiological signals derived from white matter and cerebrospinal fluid (Fox, Snyder, Vincent, & Raichle, 2007); and (c) a global signal regressor (GSR) (Fox, Zhang, Snyder, & Raichle, 2009; Murphy, Birn, Handwerker, Jones, & Bandettini, 2009). These strategies are often combined with temporal censoring of bad volumes that contain too much noise (Power, Barnes, Snyder, Schlaggar, & Petersen, 2012; Power et al., 2014; Satterthwaite et al., 2013). Unfortunately, there has been no agreement on which strategy is superior to reduce the noise present in rs‐fMRI images (Caballero‐Gaudes & Reynolds, 2017). Therefore, we investigated to which degree reproducibility of graph measures depends on the denoising techniques.

Besides the challenge of reducing noise before network construction, network construction itself can influence the reproducibility of graph measures (Dimitriadis, Drakesmith, et al., 2017; Wang et al., 2011, 2014). However, this has not been systematically explored. Network construction consists of defining the nodes of the network and defining the connectivity measure and how to calculate the weights of the network from this measure (Rubinov & Sporns, 2010). Nodes are the main elements of a network. They can be defined using an atlas or parcellation (Desikan et al., 2006; Shen, Tokoglu, Papademetris, & Constable, 2013; Tzourio‐Mazoyer et al., 2002). They can also be defined based on a set of a priori regions (Fritz et al., 2019) or based on a data‐driven method (de Vos et al., 2018) or even be defined as the voxels themselves (Horn et al., 2019). Different definitions of nodes have different advantages and limitations (Arslan et al., 2018). Node definition may affect reproducibility but also the way functional connectivity between nodes is calculated (Liang et al., 2012). The latter is typically based on a Pearson correlation but also partial correlations can be used since it can remove the influences of other nodes (Marrelec et al., 2006; Smith et al., 2011). Both types of correlation can be used to derive graphs. However, a choice needs to be made how to treat negative (partial) correlations. One option is to use the absolute value representing the information shared between two nodes. Graphs based on the absolute value are graphs in which all functional information among nodes is presented. An alternative is to study graphs based on only the positive or negative (partial) correlations separately setting the other values to zero. However, the proportion of positive and negative connections in a real network may vary, which may induce some bias if we would take only either positive correlations or negative correlations into account. The effect of these choices on the reproducibility of graph measures has not yet been systematically investigated. The last element of network construction is the choice between a weighted and a binary network. In weighted networks, a selection of connections can be made, for example, using a soft‐thresholding removing small weights and keeping the weights of the remaining connections or they can be used as fully weighted graphs in which no a priori selection is made (Li, Xue, Ellmore, Frye, & Wong, 2014; van den Heuvel, Mandl, Stam, Kahn, & Hulshoff Pol, 2010; Wang, Ghumare, Vandenberghe, & Dupont, 2017). For weighted networks, the weight can be obtained either from the raw (partial) correlations itself or from a transformed *Z*‐value after a Fisher *r*‐to‐*Z* transform (Wang et al., 2017). In binary networks, selection of connections is based on significance or amplitude. Selected connections will get a weight of 1 while the weight of the others is set to 0 (Hosseini et al., 2013; Wang et al., 2014).

Most studies only focused on the robustness of resting‐state fMRI based graph measures at the group level (Braun et al., 2012; Du et al., 2015; Paldino, Chu, Chapieski, Golriz, & Zhang, 2017). Although it can provide interesting information about brain functioning in normal or pathological conditions, it is not sufficient if we want to introduce these techniques in a clinical setting in which subject‐specific graphs have to be constructed and quantified (Gordon et al., 2017; Poldrack et al., 2015). Therefore, we focused on subject‐specific graph measures in this paper.

In this study, we comprehensively investigated how these factors mentioned above affect the reproducibility of subject‐specific graph measures and investigated which combination gave reasonable results. We investigate the use of two denoising pipelines: a simple versus a more complex denoising pipeline (Ciric et al., 2017; Parkes et al., 2018). Furthermore, we investigated the use of the two functional connectivity measures (correlations vs. partial correlations), three different approaches to handle negative correlations (taking the absolute value, taking only positive values, or taking only the negative values), two types of network (binary or weighted), and two types of calculations of the weights of the network (based on the (partial) correlation values or based on a transformation of *Z*‐values obtained after a Fisher *r*‐to‐*Z* transformation). Finally, we also considered two levels of granularity for the whole‐brain parcellation (50 vs. 100 parcels per hemisphere). The overall aim was to answer the following questions: (a) are subject‐specific graph measures reproducible and (b) what is the optimal pipeline for rs‐fMRI based subject‐specific graph measures?

## 2 MATERIALS AND METHODS

### 2.1 Dataset and ethical statement

We used multi‐session fMRI datasets from the Brain Genomics Superstruct Project (GSP) (Holmes et al., 2015). This dataset consisted of 69 healthy young adults (34 males) between 19 and 27 years old and is publically available at http://neuroinformatics.harvard.edu/gsp/. All participants provided written informed consent in accordance with guidelines established by the Partners HealthCare Institutional Review Board and the Harvard University Committee on the Use of Human Subjects in Research (Holmes et al., 2015). All images were acquired at Harvard University and Massachusetts General Hospital using Siemens 3T MAGNETOM Tim Trio MRI scanners equipped with a 12‐channel phase‐array head coil. Each subject had two sessions on a different day with a gap between scan days within 6 months. Each session consisted of a structural scan and one or two runs of resting‐state fMRI. The structural image was a T1‐weighted Multi‐Echo MPRAGE (ME‐MPRAGE) image with 1.2 mm isotropic resolution (van der Kouwe, Benner, Salat, & Fischl, 2008). The BOLD images had a 3 mm isotropic resolution. Each BOLD volume had 47 slices including the full cerebellum. The interleaved slices were acquired in an interleaved fashion in ascending order (from bottom to top). The TR was 3 s, and the number of volumes in each run was 124. The total scan time of each functional run was 6 min and 12 s. During BOLD data collection, all participants were instructed to keep their eyes open while blinking normally. For more details about the scans, see (Holmes et al., 2015).

### 2.2 Standard preprocessing

The standard preprocessing was performed using Matlab (R2014b) and Statistical Parametric Mapping software (version SPM12, Wellcome Department of Cognitive Neurology, London, UK; http://www.fil.ion.ucl.ac.uk/spm/software/spm12/; RRID:SCR_007037). Before data preprocessing, we set the origin for all structural and functional MRI images close to the anterior commissure and if needed, adapted the orientation to make it more similar to the template in MNI space. This step only affected the transformation matrix and not the data itself since we did not reslice the images. The standard preprocessing steps included: (1) The first four dummy scans of each run were removed; (2) all resting‐state functional images were realigned to correct for head movement; (3) slice timing; (4) coregistration of the structural and mean functional image; (5) segmentation of the structural image which provides deformation fields to warp the data to MNI space; and (6) warping of the functional images to MNI space using the deformation field of each subject obtained in the previous step.

### 2.3 Image quality control

Besides visually checking image quality by a neuroradiologist (QR) to make sure there were no apparent structural abnormalities or artifacts present, image quality of the rs‐fMRI data was further assessed using the head movement parameters obtained during the realignment step (overall translation or rotation along any direction and framewise displacement) and the change in brain intensities between two consecutive volumes (Power et al., 2012, 2014).

Framewise displacement (FD) was calculated as (Power et al., 2012, 2014).

$${\text{FD}}_{i}=\left|\Delta d_{\mathrm{ix}}\right|+\left|\Delta d_{\mathrm{iy}}\right|+50*\left(\left|\Delta d_{\mathrm{iz}}\right|+\left|\Delta \alpha_{i}\right|+\left|\Delta \beta_{i}\right|+\left|\Delta \gamma_{i}\right|\right)$$

in which $\Delta d_{\mathrm{ix}}=d_{\left(i-1\right)x}-d_{\mathrm{ix}}$, and similarly for the other movement parameters $\left[d_{\mathrm{ix}}\;d_{\mathrm{iy}}\;d_{\mathrm{iz}}\;\alpha_{i}\;\beta_{i}\;\gamma_{i}\right]$. Note that the rotation parameters should be expressed in radians and translation parameters in mm.

The volume by volume intensity changes across whole‐brain voxels are calculated as (Power et al., 2012; Smyser et al., 2010).

$$\text{DVARS}{\left(\Delta I\right)}_{i}=\sqrt{〈\left[\Delta I_{i}\left(\vec{x}\right)\right]〉}=\sqrt{〈{\left[I_{i}\left(\vec{x}\right)-I_{i-1}\left(\vec{x}\right)\right]}^{2}〉}$$

in which $I_{i}\left(\vec{x}\right)$ is the image intensity at locus $\vec{x}$ in volume i, <.> denotes the spatial average over the whole‐brain voxels (Power et al., 2012; Smyser et al., 2010).

In this work, we used a cutoff value for FD of <0.5 mm consistent with values reported by Waheed et al. (2016). The DVARS threshold was set (per run) at the mean of the DVARS values over time plus three standard deviations. The overall translation (across the run) was required to be <1.5 mm and the overall rotation <1.5 degrees (in all directions). When no 5 min interval was present with these characteristics, we applied scrubbing on those volumes which were outside these predefined limits. We only did this for at most 20 “bad” volumes. Scrubbing seems compelling to reduce head movement effects but at the cost of loss of temporal degrees of freedom (Ciric et al., 2017; Parkes et al., 2018). Furthermore, if too many volumes required scrubbing, it is not clear if the scrubbed data still represent the underlying connectivity. When none of the above was satisfied, we considered the data as of too low quality and that run was taken off‐study.

To investigate the reproducibility of graph measures among sessions, we selected the first run of each session except when the first run was taken off‐study in which case the second run was used.

### 2.4 Pipelines for denoising

We selected two denoising pipelines: a simple approach on the one hand and a complex one on the other hand to evaluate the effect of denoising on the reproducibility of graph measures. The simple approach consisted of the inclusion of 9 regressors: 6 head motion parameters, 2 physiological parameters (a WM and CSF regressor, see below), and the GSR. The complex denoising pipeline had 30 regressors: 24 head motion parameters (6 basic ones along with their temporal derivatives, squared values and the squared values of the temporal derivatives), 2 physiological parameters along with their temporal derivatives and the global signal regressor along with its derivative.

The head motion parameters were derived from the realignment step in the preprocessing. WM and CSF regressors were extracted in a WM and a CSF mask, respectively, which were calculated as the intersection between the subject‐specific WM and CSF segmentations (thresholded at 0.9) and a priori masks of WM and CSF. The a priori masks were generated from the a priori masks from SPM (in case of CSF, we limited it to the lateral ventricles) by thresholding them at 0.5 and by applying an erosion to avoid being too close to GM.

The global signal regressor was calculated as the average BOLD time series across all voxels within a global brain mask defined as voxels in which the sum of the GM, WM, and CSF segmentation maps is more than 0.9 (Fox et al., 2007; Shirer, Jiang, Price, Ng, & Greicius, 2015). We included GSR in both simple and complex denoising pipelines.

In case scrubbing was required (i.e., when censored volumes were identified), the censored volumes were not taken into account in any of the preprocessing steps except for the band‐pass filtering in which case they were marked, and their values were replaced by linear interpolation before the filtering. During the calculation of the functional connectivity, the censored volumes were not used. It is worth mentioning that we applied scrubbing of bad volumes in both denoising pipelines (Power et al., 2014) if required.

After defining all regressors, we applied a principal component analysis (PCA) to avoid multi‐collinearity of regressors (Jackson, 1991).

After removal of the nuisance signals, band‐pass filtering was applied (0.009–0.1 Hz).

### 2.5 Network construction

A network consists of nodes and connections. The nodes in this study were derived from a whole‐brain parcellation using different levels of granularity (Shen et al., 2013). We selected 50 parcels (Shen50) and 100 parcels (Shen100) per hemisphere for this work (https://www.nitrc.org/frs/?group_id=51). Each parcel was taken as a node of the network.

For each subject, the average time series of each parcel was calculated as the mean of the time series over all voxels in the parcel. Based on the average time series, we calculated either a Pearson correlation or a partial correlation between the average time series in any pair of parcels. The partial correlations were calculated between any pair of time series taking the other time series as controllers. The calculation was based on the inversion of the covariance matrix. Partial correlations remove the influence of other regions and could be considered as a more specific measure for functional connectivity (Marrelec et al., 2006; Smith et al., 2011).

We studied two types of networks: a binary network and a weighted undirected network without self‐connections. Binary networks were studied at different densities. The density should neither be too sparse nor too dense (Hosseini et al., 2013; Kaiser & Hilgetag, 2006). Therefore, we used densities ranging from 5% to 40% (Wang et al., 2014). A weighted network can avoid the problem of thresholding and takes all weights into account (Schwarz & McGonigle, 2011; Wang et al., 2011, 2017; van Wijk, Stam, & Daffertshofer, 2010). The weights have a value between 0 and 1, and they were calculated in different ways: (partial) Correlations were directly taken as weights by either (a) its absolute value (abs), (b) by taking only the positive values and setting negative values to 0 (pos), or (c) by setting positive values to zero and then take the absolute value of the negative values (neg).

Another way is to transform (partial) correlations to *Z*‐scores using a Fisher *r*‐to‐*Z* transform (Finn, 1974):

$$Z=\frac{\sqrt{n-3-\left(p-2\right)}}{2}\ln\frac{1+r}{1-r}$$

in which r indicates the (partial) correlation, *n* is the number of volumes, and *p* is the number of regions (which is 2 in case of correlations) (Finn, 1974; Wang et al., 2014). Weights are then calculated from the *Z*‐scores as (Wang et al., 2017).

$$w={\left\{2\Phi \left(\left|Z\right|\right)-1\right\}}^{4}$$

in which Φ is the cumulative distribution function of the standard normal distribution.

Similar as before, one can take all the *Z*‐values (abs), set the negative Z‐values to zero (pos), or set the positive *Z*‐values to zero (neg) before calculating the weights *w*. Although the approach in which we calculate weights based on *Z*‐scores is theoretically better, it suffers from two problems: The number of volumes should be greater than the number of parcels plus one in order to obtain real values when calculating partial correlations directly without any regularization approach. In the current dataset, the maximally available number of volumes was 120 for a whole run. Therefore, partial correlations were only obtained for the first level of granularity (50 parcels per hemisphere). The other problem is that the values of the *Z*‐scores depend on the number of volumes used, and this has an impact on the graph measures themselves. This can be a problem when combining data from subjects with a different number of volumes.

### 2.6 Graph analysis

Graph theoretical analysis was conducted using the brain connectivity toolbox version 2017‐05‐01 (https://sites.google.com/site/bctnet/; RRID:SCR_018421; Rubinov & Sporns, 2010). We calculated nodal and global measures. The nodal graph measures were average path length, nodal clustering coefficient, nodal efficiency, and nodal betweenness centrality. The global graph measures included characteristic path length, clustering coefficient, efficiency, and betweenness centrality. These graph measures were calculated at the individual level. Furthermore, hubs were defined based on the hub score. The hub score is calculated as the sum of dummy values (0 or 1) for four criteria based on whether the node belongs to the top 20% of nodes (a) showing the highest degree, (b) showing the lowest path length, (c) showing the lowest clustering coefficient, and (d) showing the highest betweenness centrality. If the hub score was at least 2, the node was considered a hub. The modularity structure was determined using Newman's algorithm using 100 realizations (Vandenberghe et al., 2013; Wang et al., 2014). This defines the probabilistic co‐assignment matrix in which the probability is given that two nodes belong to the same module when applying Newman's algorithm. We also calculated normalized graph measures. A normalized graph measure is the ratio between the graph measure and the average graph measure of 30 equivalent random networks. Equivalent random networks have the same number of nodes and the same density (in case of binary networks) or the same distribution of connectivity values (for a weighted network) but connections are randomly assigned to each pair of nodes.

### 2.7 Measures of reproducibility for graph measures

The reproducibility of graph measures (Wang et al., 2014) at the individual level can be easily assessed using the test–retest variability (TRT) (in %):

$$\text{TRT}=100\left|\frac{m_{2}-m_{1}}{\frac{m_{1}+m_{2}}{2}}\right|$$

$m_{1}$ and $m_{2}$ are the values of the graph measure derived from the first and second measurement, respectively. A higher TRT value indicates higher variability and less reproducibility.

The consistency of hubs is determined by measuring the co‐occurrence (Hc) of hubs (Wang et al., 2014) which is based on the Dice coefficient.

$$\text{Hc}=\frac{2\left|H_{1}\cap H_{2}\right|}{\left|H_{1}\right|+\left|H_{2}\right|},$$

where *H_1_* and *H_2_* are the list of hubs in the network of the first, respectively, and second measurements.

A value of 1 corresponds to a perfect agreement of hubs while 0 reflects no agreement at all.

To assess the consistency of the modularity structure, we used probabilistic scaled inclusivity (pSI) (Wang et al., 2014):

$$pSI\left(i\right)=\frac{{\left(\sum_{j=1}^{N}P_{1}\left(i,j\right).P_{2}\left(i,j\right)\right)}^{2}}{\sum_{j=1}^{N}P_{1}\left(i,j\right).\sum_{j=1}^{N}P_{2}\left(i,j\right)}$$

Here, i and j indicate two nodes; *P_1_(i,j)* and *P_2_(i,j)* are the probabilistic co‐assignment between nodes i and j in the network derived from the first and second measurement, respectively. A value of 1 corresponds to a perfect agreement of modularity while 0 reflects no agreement at all. Here, we calculated pSI for each node.

### 2.8 Statistics

In this study, we investigate the effect of different factors on the reproducibility of graph measures. These factors included denoising pipelines (simple vs. complex), types of correlation (Pearson correlation vs. partial correlation), the handling of negative correlation values (absolute vs. positive vs. negative values), type of network (weighted vs. binary networks), the weight calculation (based on the (partial) correlation or based on a Fisher *r*‐to‐*Z* based transformation), and the granularity of the parcellation (50 vs. 100 parcels per hemisphere). To ensure that the results are not depending on the weight distribution or the density, all comparisons for weighted networks were based on normalized graph measures, and for binary networks, we used identical densities.

Most graph measures were not normally distributed (based on a Shapiro–Wilk test), and therefore, we used a relative nonparametric analysis of variance through the aligned rank transform method (ART) (Wobbrock, Findlater, Gergle, & Higgins, 2011).

The analyses were performed in three steps.

First, we used a 2 × 2 factorial repeated measures nonparametric analysis of variance using ART to assess TRT values of graph measures, in which the first factor was the denoising pipeline (simple vs. complex denoising) and the second factor was the type of correlation (Pearson correlation vs. partial correlation). For this analysis, we used the Shen50 parcellation and the absolute value of the (partial) correlations. We performed the analysis separately for binary networks at different densities and for weighted networks. In the latter case, we introduced a third factor based on how the weights were calculated (using the (partial) correlation values or using weights based on the Fisher *r*‐to‐*Z* transformation), and thus, we analyzed a 2 × 2 × 2 factorial repeated measures nonparametric analysis of variance.

Second, we performed a 3 × 2 factorial repeated measures nonparametric analysis of variance using ART (ARTool in R) in which the first factor was the handling of the negative correlations (absolute value, only positive values, and only negative values) and the second factor was the type of correlation (Pearson correlation vs. partial correlation). For this analysis, we used—based on the results of the first analyses (see below)—the Shen50 parcellation, the simple denoising pipeline, and weighted graphs in which weights were calculated based on the value of the (partial) correlations.

Third, we used ART to compare the effect of the level of parcellation (50 vs. 100 parcels per hemisphere). In this analysis, we used again the simple denoising pipeline and weighted graphs in which weights were calculated based on the value of the correlations. We also had to limit this analysis to Pearson correlations since partial correlations cannot be reliably calculated given the number of data points in the time series compared to the number of nodes.

**FIGURE 1:**
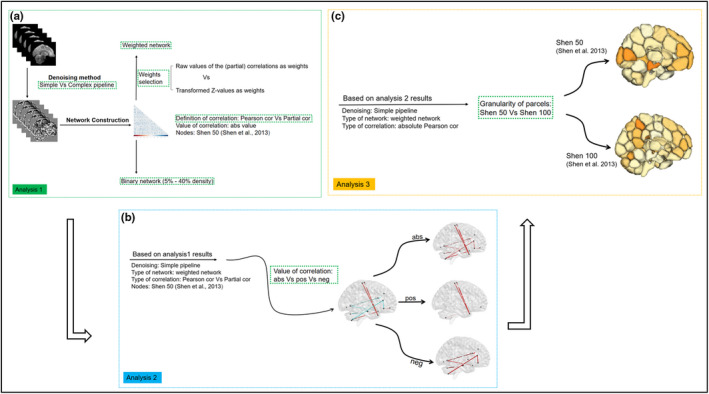
Flow chart of the different analyses

An overview of all analyses is given in Table 1 and Figure 1.

**TABLE 1:** Statistical analyses

*Analyses 1*: Factorial repeated measures nonparametric analysis of variance using the Shen50 whole‐brain parcellation and using absolute values of the (partial) correlations
*1a binary networks*: 2 × 2 design with *factor 1*: denoising pipeline (simple vs. complex) and *factor 2*: type of correlation (Pearson correlation vs. partial correlations) for different densities
*1b weighted networks*: 2 × 2 × 2 design with *factor 1*: denoising pipeline (simple vs. complex); *factor 2*: type of correlation (Pearson correlation vs. partial correlations); and *factor 3*: weight calculation method (based on the raw values of the (partial) correlations vs. based on transformed Z‐values obtained after a Fisher *r*‐to‐Z transform)
*Analysis 2*: 3 × 2 factorial repeated measures nonparametric analysis of variance using the Shen50 whole‐brain parcellation using the simple denoising pipeline and weighted graphs in which the weights are based on the values of the (partial) correlations. *Factor 1*: handling of the negative correlations (absolute value, only positive values, and only negative values) and *factor 2*: type of correlation (Pearson correlations vs. partial correlations)
*Analysis 3*: Wilcoxon signed rank test between the normalized graph measures obtained using two different whole‐brain parcellations (Shen50 vs. Shen100) in which 50 and 100 refer to the number of parcels per hemisphere. We used a simple denoising strategy, absolute values of the correlation and weighted networks in which weights were calculated based on the raw values of the correlation

The *p*‐value was set at a Bonferroni corrected *p* < .05 to take into account multiple comparisons (the number of graph measures we have evaluated although graph measures are not completely independent). We did not correct for the number of analyses we performed, to prevent too much reduction of power. All statistical analyses were performed in Rstudio (version 3.6.0).

## 3 RESULTS

### 3.1 The effect of denoising pipelines and types of correlation

#### 3.1.1 Binary networks

**FIGURE 2:**
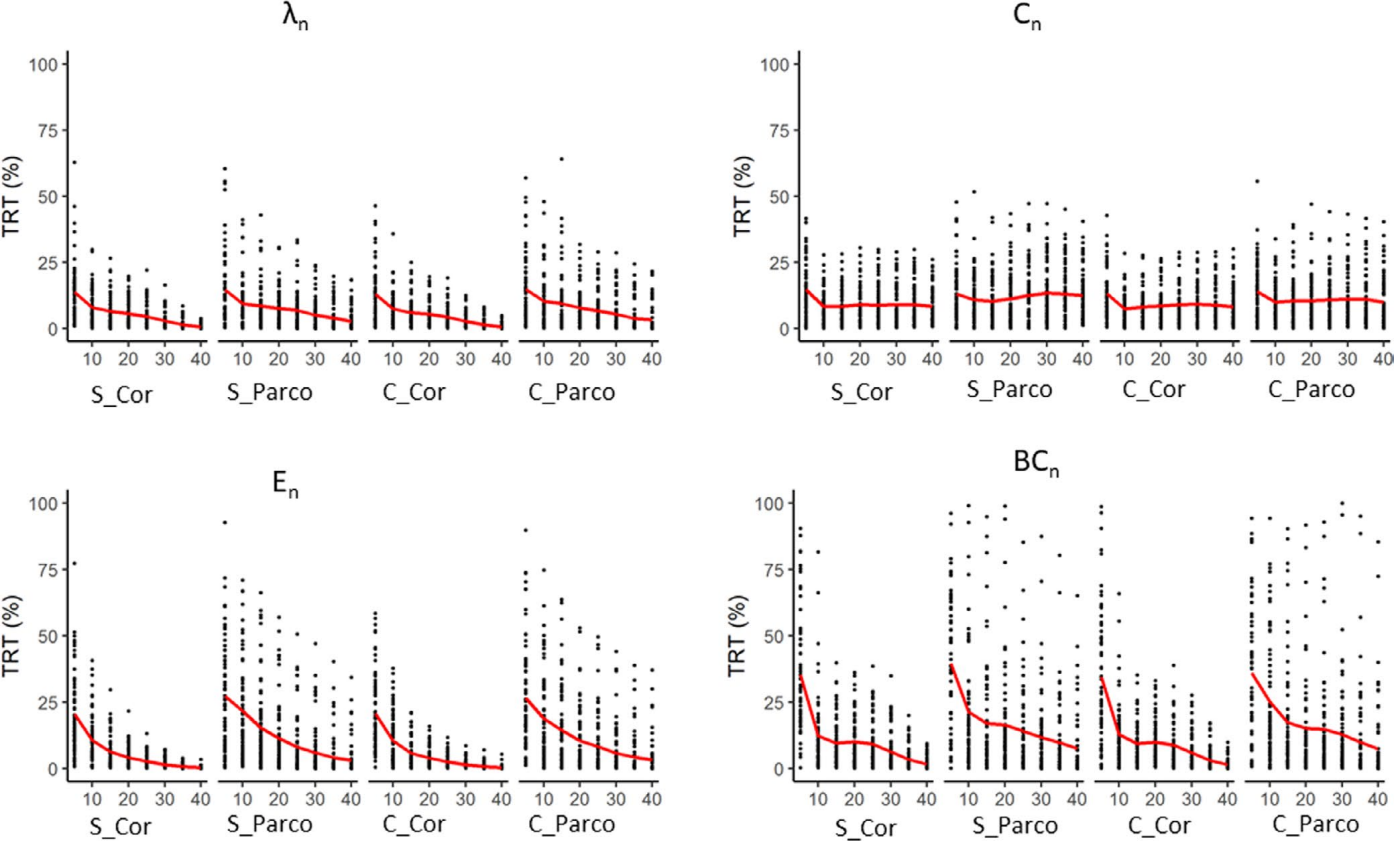
TRT (%) values of global graph measures in binary networks. The *x*‐axis indicates density (in percent) of the network ranging from 5% to 40%. S, simple denoising; C, complex denoising; Cor, Pearson correlation; Parco, partial correlation; *λ*_n_, normalized characteristic path length; *C*_n_, normalized clustering coefficient; *E*_n_, normalized efficiency; and BC_n_, normalized betweenness centrality

In binary networks, the TRT variability for global measures was large at low density (Figure 2, Table S1) irrespective of the denoising strategy or the type of correlation. Comparing the different strategies (analysis 1a, Table 1; Figure 1a), the type of correlation (Pearson correlation vs. partial correlation) has a significant effect on TRT (Table 2): Pearson correlations showed lower TRT values (Table S1). The denoising pipelines (simple vs. complex denoising) did not have a significant effect, and there was no interaction effect between the denoising pipelines and the type of correlation (Table 2). Since TRT values were large at lower densities even for global graph measures (Table S1), we did not further investigated binary networks.

**TABLE 2:** Comparison of denoising strategy and type of correlation on test–retest values for global graph measures of binary networks (analysis 1a)

Graph measure	Density (%)	*df*	Main effects				Interaction	
			Denoising (Simple vs. Complex)		Correlation (Pearson correlation vs. partial correlation)		Denoising × Correlation	
			*F*	*p*	*F*	*p*	*F*	*p*
*λ_n_*	5	(1,204)	0.0	.86	0.0	.96	0.5	.48
	10	(1,204)	0.0	.85	2.1	.15	0.3	.59
	20	(1,204)	0.3	.60	10.1	**1.7E‐03**	0.1	.78
	30	(1,204)	0.2	.63	9.9	**1.9E‐03**	0.6	.42
	40	(1,204)	0.9	.33	27.2	**4.4E‐07**	0.2	.66
*C_n_*	5	(1,204)	0.0	.98	0.7	.41	4.1	*.04*
	10	(1,204)	0.1	.72	5.0	**2.6E‐02**	0.3	.58
	20	(1,204)	0.5	.47	1.8	.18	0.0	.86
	30	(1,204)	1.2	.27	6.1	**1.4E‐02**	1.9	.17
	40	(1,204)	3.8	.05	7.3	**7.5E‐03**	3.4	.07
*E_n_*	5	(1,204)	0.0	.91	5.3	**2.2E‐02**	0.1	.73
	10	(1,204)	2.0	.15	36.6	**6.7E‐09**	1.7	.20
	20	(1,204)	0.8	.38	25.9	**8.2E‐07**	0.6	.42
	30	(1,204)	0.1	.73	19.9	**1.3E‐05**	0.0	.89
	40	(1,204)	2.4	.12	30.0	**1.3E‐07**	1.5	.22
BC_n_	5	(1,204)	0.7	.41	5.5	**2.0E‐02**	0.1	.71
	10	(1,204)	1.7	.20	28.8	**2.2E‐07**	0.3	.58
	20	(1,204)	0.1	.78	4.0	**4.7E‐02**	0.2	.68
	30	(1,204)	0.0	.99	10.6	**1.3E‐03**	0.1	.78
	40	(1,204)	0.2	.65	22.1	**4.7E‐06**	0.0	.97

Note

*p*‐values are uncorrected but those in bold are significant after a Bonferroni correction (*p* < .05) correcting for 4 tests.

#### 3.1.2 Weighted networks

**FIGURE 3:**
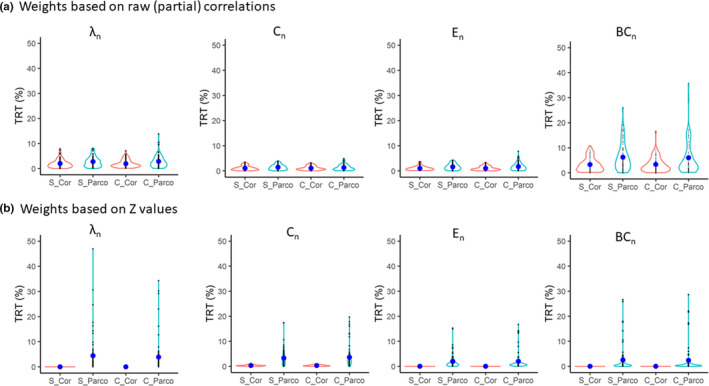
TRT (%) values of global graph measures in weighted networks. (a) weights are based on the raw (partial) correlations; (b), weights are based on Fisher's transformed *r*‐to‐*Z* values. BC_n_, normalized betweenness centrality; C, complex denoising; *C*_n_, normalized clustering coefficient; Cor, Pearson correlation; *E*_n_: normalized efficiency; Parco, partial correlation; S, simple denoising; *λ*_n_, normalized characteristic path length

Weighted networks showed very low TRT values for global graph measures (Figure 3, Table 3A, Table S2) irrespective of the strategy used. Comparing the TRT values for different strategies (analysis 1b, Table 1, Figure 1a), there were significant main effects of the type of correlation (Pearson correlation vs. partial correlation) and weights calculation (based on the raw (partial) correlation values vs. based on transformed *Z*‐values) (Table 3B). There was also a significant interaction effect between type of correlation and weight calculation (Table 3B). Similarly as for the binary networks, there was no main effect of the denoising pipeline.

**TABLE 3:** Summary of normalized global graph measures for weighted networks (analysis 1b). A. Results for the simple denoising strategy when using the raw partial correlations as weight. B. Results of the statistical analysis

A								
	Correlations				Partial correlations			
	Values at time point 1		TRT (%)		Values at time point 1		TRT (%)	
	Mean	*SD*	Mean	*SD*	Mean	*SD*	Mean	*SD*
*λ*_n_	1.121	0.017	2.0	1.7	1.125	0.030	2.7	2.0
*C*_n_	1.032	0.009	1.1	0.9	1.036	0.013	1.4	1.0
*E*_n_	0.929	0.007	1.0	0.8	0.930	0.012	1.6	1.2
BC_n_	0.921	0.023	3.3	2.8	0.916	0.047	6.2	5.4

B							
Main Effects	*df*	Denoising (S vs. C)		Correlation (Cor vs. Parco)		Weight (R vs. Z)	
		*F*	*p*	*F*	*p*	*F*	*p*
λ_n_	(1,476)	5.5	.02	122.4	**<1.0E‐15**	23.7	**1.50E‐06**
*C*_n_	(1,476)	0.6	.42	241.1	**<1.0E‐15**	22.5	**2.80E‐06**
*E*_n_	(1,476)	0.5	.49	126.8	**<1.0E‐15**	53.4	**1.20E‐12**
BC_n_	(1,476)	5.5	.02	129.2	**<1.0E‐15**	413.7	**<1.0E‐15**

Interactions	*df*	Denoising × correlation		Denoising × weight		Correlation × weight		Denoising × correlation × weight	
		*F*	*p*	*F*	*p*	*F*	*p*	*F*	*p*
*λ*_n_	(1,476)	4.7	.032	3.0	.08	43.5	**1.10E‐10**	4.1	.04
*C*_n_	(1,476)	0.6	.43	0.5	.47	162.8	**<1.0E‐15**	0.5	.48
*E*_n_	(1,476)	0.6	.45	3.7	.05	11.8	**6.50E‐04**	1.1	.28
BC_n_	(1,476)	5.4	.021	0.4	.55	13	**3.50E‐04**	5.9	.02

Note

*p*‐values are uncorrected but those in bold are significant after a Bonferroni correction (*p* < .05) correcting for 4 tests.

**FIGURE 4:**
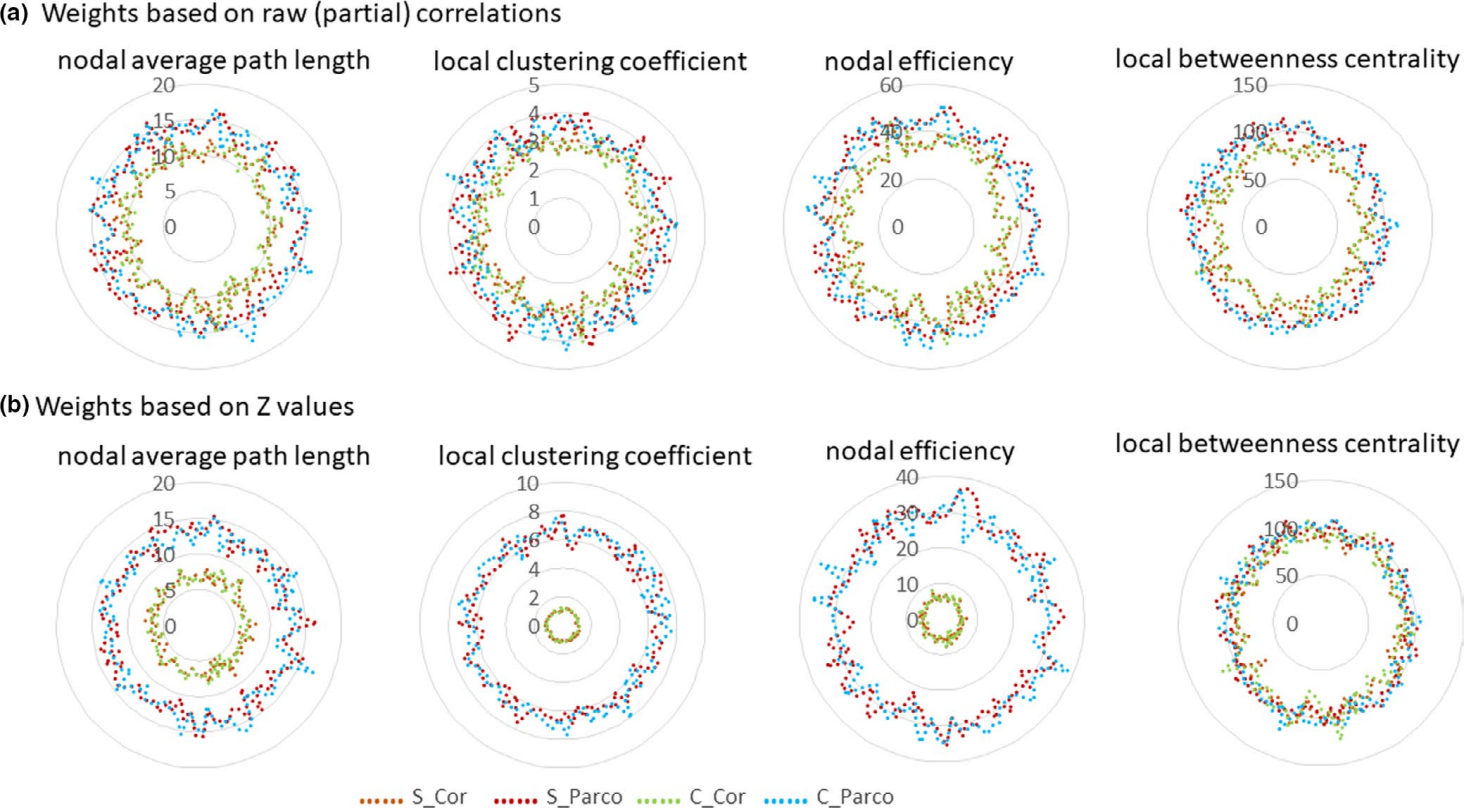
TRT (%) values of nodal graph measures based on different weight calculations. (a) weights are based on the raw (partial) correlations; (b) weights are based on Fisher's transformed *r*‐to‐*Z* values. Each node is located on a circle and the radius represents the TRT value. C, complex denoising; Cor, Pearson correlation; Parco, partial correlation; S, simple denoising

Local clustering coefficient and nodal average path length showed relatively low TRT values but local betweenness centrality showed high TRT values (Figure 4, Table S3). Nodal efficiency also showed high TRT values in all cases except when weights were calculated based on transformed *Z*‐values of the correlations.

Hub consistency was moderate with average values (for the different strategies) between 0.23 and 0.29.

The average consistency of the modularity structure was between 0.18 and 0.24.

#### 3.1.3 Binary and weighted networks constructed using orthogonal minimal spanning trees

We have also applied a data‐driven topological filtering approach based on orthogonal minimal spanning trees (OMST) to construct a binary and a weighted network. This method was recently proposed by Dimitriadis, Antonakakis, Simos, Fletcher, and Papanicolaou (2017), Dimitriadis, Salis, Tarnanas, and Linden (2017). The results are given in Tables S5 and S6 and are in line with the results obtained before.

#### 3.1.4 Binary and weighted networks constructed using the Oxford–Harvard atlas

To ensure that the results were not critical depending on the Shen50 atlas, we also performed an additional analysis using the Oxford–Harvard atlas (Desikan et al., 2006; Frazier et al., 2005; Goldstein et al., 2007; Makris et al., 2006). The results (shown in Tables S7‐S10) were in the same line as for the Shen50 atlas.

### 3.2 The effect of type of correlation and the handling of negative values

In a second analysis (analysis 2, Table 1, Figure 1b), we investigated the TRT variability of different strategies to handle negative values in the (partial) correlations (see Section 2). In this analysis, we used the Shen50 parcellation to define the nodes and the simple denoising pipeline according to the outcome of the first analysis.

**FIGURE 5:**
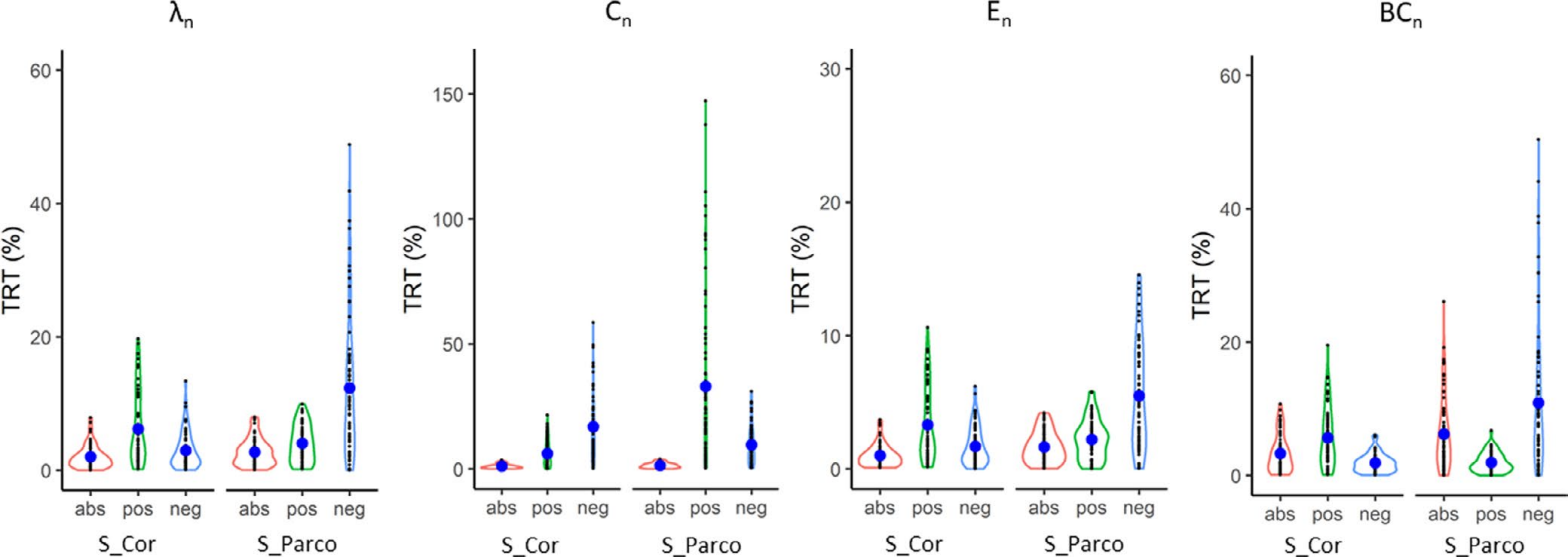
TRT (%) values of global graph measures based on different (partial) correlation value: handling of negative values. Abs: values based on the absolute (partial) correlations; BC_n_, normalized betweenness centrality; C, complex denoising; *C*_n_, normalized clustering coefficient; Cor, Pearson correlation; *E*_n_, normalized efficiency; neg, values based on only the positive (partial) correlations; Parco, partial correlation; pos, values based on only the positive (partial) correlations; S, simple denoising; *λ*_n_, normalized characteristic path length

There was a significant main effect of the handling of negative values in which taking the absolute value showed overall the lowest test–retest values (Figure 5, Table S4). Similar to analysis 1, there was a significant main effect of the type of correlation, in which Pearson correlation‐based normalized global graph measures showed lower test–retest values compared to the ones based on partial correlations (Table S4). There was also a significant interaction effect (Table S4).

Hub consistency ranged from 0.22 to 0.29, and the average consistency of the modularity structure was between 0.17 and 0.26.

### 3.3 The effect of parcellation granularity

In the third analysis (analysis 3, Table 1, Figure 1c), we used the simple denoising pipeline and weighted graphs in which weights were calculated based on the absolute value of the (partial) correlations. We only varied the number of parcels per hemisphere: 50 versus 100 by comparing the results for the Shen50 versus Shen100 parcellation.

**FIGURE 6:**
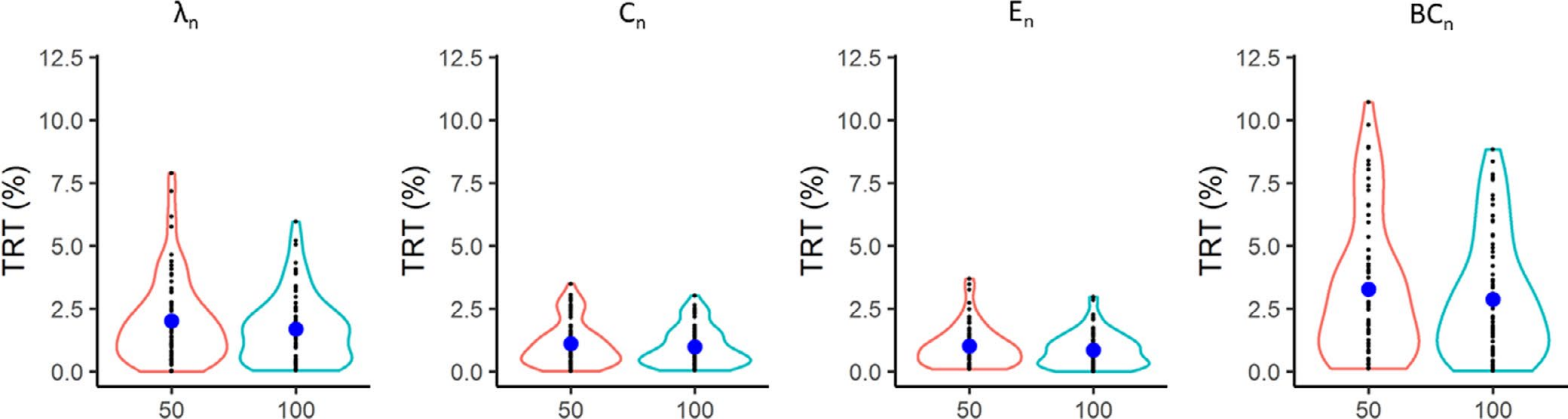
TRT (%) values of global graph measures based on different granularity. The *x*‐axis indicates the level of granularity. 50 and 100 relate to the 50, respectively, and 100 parcels per hemisphere used in the brain parcellation. BC_n_: normalized betweenness centrality; *C*_n_: normalized clustering coefficient; *E*_n_: normalized efficiency; *λ*_n_: normalized characteristic path length

Mean TRT values of all global graph measures obtained using the Shen100 parcellation decreased compared to the values obtained using the Shen50 parcellation (Table 4A). However, the difference was not significant (all *p*‐value > .05) (Table 4B). The distribution of TRT for all global graph measures for the Shen50 and Shen100 parcellation can be found in Figure 6.

**TABLE 4:** Normalized global graph measures for different levels of granularity and their comparison (analysis 3)

A								
	Shen50				Shen100			
	Values at time point 1		TRT (%)		Values at time point 1		TRT (%)	
	Mean	*SD*	Mean	*SD*	Mean	*SD*	Mean	*SD*
λ_n_	1.121	0.017	2.0	1.7	1.129	0.015	1.7	1.4
*C*_n_	1.032	0.009	1.1	0.9	1.031	0.008	1.0	0.8
*E*_n_	0.929	0.007	1.0	0.8	0.922	0.006	0.9	0.7
BC_n_	0.921	0.023	3.3	2.8	0.920	0.023	2.9	2.4

B			
	Parcellation (50 vs. 100)		
	*df*	*F*	*p*
*λ*_n_	(1,68)	3.3	.073
*C*_n_	(1,68)	3.7	.058
*E*_n_	(1,68)	1.8	.189
BC_n_	(1,68)	2.3	.133

Note

*p*‐values are uncorrected.

## 4 DISCUSSION

In this study, we investigated the effects of denoising pipelines and network constructions on reproducibility of graph measures. We found that the choice of denoising pipeline did not significantly affect the reproducibility of graph measures. Furthermore, the reproducibility of graph measures of individual *binary* networks was insufficient, especially when the network density was low. This was also the case for the reproducibility of nodal graph measures, in particular local betweenness centrality and nodal efficiency. For *weighted* networks, the method of choice was the absolute value of the (partial) correlation as weight. The reproducibility of normalized global graph measures did not critically depend on the level of granularity or the parcellation scheme that was used.

### 4.1 Denoising: simple denoising versus complex denoising

We used a simple and a complex denoising strategy by means of a model with a low or a high number of parameters. The parameters of the simple model included the six basic head movements and regressors for WM, CSF, and a GSR. This pipeline is widely applied for preprocessing of functional connectivity studies (Fox et al., 2009). The parameters of the complex model consisted of the same parameters as the simple model but extended with their temporal derivatives, their squared values and the squared values of the temporal derivatives (Ciric et al., 2017, 2018; Parkes et al., 2018). According to the findings of Parkes et al. (2018), the percentage of edges significantly associated with head motion was 10.7% for the simple denoising strategy versus 10.4% for the complex denoising strategy (Parkes et al., 2018) which is very similar. In our study, we also found that the reproducibility of the graph measures of networks preprocessed using either the simple or complex denoising strategy was very similar and statistically not different.

Including the GSR, dominated by non‐neural signals such as motion‐related and respiratory noise rather than signals from regions of gray matter, has been demonstrated to noticeably decrease the impact caused by head movement‐related artifacts and other physiological confounds (Power, Plitt, Laumann, & Martin, 2017). It had also been reported that including the GSR could introduce negative correlations (Fox et al., 2009; Murphy et al., 2009) and may have an impact on graph measures (Chen et al., 2018). In this work, we included the GSR based on the work of Parkes and Ciric (Ciric et al., 2017; Parkes et al., 2018). The combination of head movement parameters and physiological non‐neural signal regressors and GSR is a relatively effective way to decrease the influence by noise. It improved the reproducibility of the functional connectivity among regions (Parkes et al., 2018). Additionally, we investigated differences in reproducibility between a simple and a complex denoising models at the subject level and in both denoising models, a global signal regressor was included.

### 4.2 Definition of correlation: correlation versus partial correlation

In this study, we found that graph measures based on Pearson correlations showed lower TRT values versus those based on partial correlations. Once nodes have been defined, another important question to consider is how to quantify the interaction among these spatially distinct regions through neurobiologically interpretable quantities. The most commonly used functional interactions are based on correlations (Kirino et al., 2019) or partial correlations (Lin et al., 2018) between the time course of two nodes. Functional connections based on correlations between two spatially distinct regions could be driven by other regions (Wang et al., 2014). Functional connections based on partial correlations avoid this caveat, and they are more related to effective connectivity (Marrelec et al., 2006; Smith et al., 2011). Therefore, they are a more appropriate method (Dawson et al., 2016; Wang, Kang, Kemmer, & Guo, 2016) to detect biologically interpretable alterations of graph measures under different disease conditions. This might also explain the higher TRT values of graph measures based on partial correlations: They could provide more biologically meaningful information and therefore are more dependent on the brain state.

### 4.3 Weight selection

We demonstrated that the weight selection affected the TRT values of graph measures. How to generate weights when applying a weighted network analysis has not been systematically investigated until now. We explored its effect on TRT of graph measures through combining it with other network construct factors. The weights were derived either using raw (partial) correlations or using *Z*‐values obtained after a Fisher *r*‐to‐*Z* transform. The weight distribution depends on this choice.

When looking at differences at the functional connectivity level, it is mandatory to use a Fisher *r*‐to‐*Z* transform in order to perform a correct statistical analysis. However, this transformation depends on the number of data points that is used. In this study, the calculation of graph measures was based on the functional connectivity. As a result, the weights when calculated using these Z‐values also depend on the number of data points. As long as all data have the same number of data points, this is not a fundamental problem; but when pooling data with a different number of data points, this approach cannot be used.

### 4.4 Binary versus weighted networks

Binary networks can be constructed independently of the method to define weights since they are typically studied at different densities and the selection of the highest connections based on raw (partial) correlations, transformed *Z*‐values, or weights is the same since these methods preserve the order of highest connections. However, we have shown that the reproducibility of binary networks at the subject level is low especially at lower densities such as 5%–10%. This is not surprising since at lower densities, each change in connection has a more dramatic impact on the graph measures. Group based binary networks are more reproducible (Wang et al., 2014) but in the current study, we focused on the use of graph measures derived from single subjects since this is the preferred method to use in a clinical setting (Xiang et al., 2019).

### 4.5 (Partial) correlation values: absolute, positive, and negative

When using (partial) correlations as measure of functional connectivity between nodes, one needs to decide how to handle negative values. In most studies, either the absolute value is used or the connectivity is limited to only those connections with a positive value while neglecting the negative (partial) correlations (Kazeminejad & Sotero, 2019). However, negative correlations may contain important biological information, which cannot be neglected (Kazeminejad & Sotero, 2019). We found that the reproducibility of graph measures for networks based on only positive or only negative values were worse compared to taking the absolute value. Furthermore, the proportion of positive and negative (partial) correlations in a given network may vary, which may induce bias if we take only either positive or negative correlations into account. Therefore, we recommend using the absolute value, because connections with negative correlations are taken into account and the absolute value can be interpreted as the amount of information shared between two regions. The only drawback is that we cannot distinguish between positive or negative correlations with the same amplitude.

### 4.6 Granularity of parcels

The first step in defining a graph is the definition of the nodes of the network. There are many different ways to define nodes such as based on cytoarchitecture, anatomy, or connectivity‐driven methods with different levels of granularity (Arslan et al., 2018).

Parcellation methods based on functional data have nodes which are more homogeneous and functionally coherent (Arslan et al., 2018). In our study, we have used a brain parcellation obtained from connectivity‐driven analyses using spectral graph theory (Shen et al., 2013). These parcellations were available for different levels of granularity, which made it easier to study the effect of granularity. Using parcellation schemes which were based on different criteria would be an extra confounding factor. Furthermore, there is currently no golden standard for which parcellations to use.

### 4.7 Hubs and modularity

The hubs and the modularity structure were only partially consistent at the subject level. This may be partially explained because of the low reproducibility of the local graph measures. Another possibility is that the identification of hubs and modules is affected by different brain states present during the resting‐state experiment (Kabbara et al., 2019). As a result, the hubs and modules may be different during different brain states which may explain the limited reproducibility. This hypothesis has to be tested by further investigations.

### 4.8 Limitations

There are a number of limitations in this study. First, we did not investigate all possible denoising pipelines, because the effect of denoising pipelines on functional connectivity has been studied previously (Ciric et al., 2017; Parkes et al., 2018). We selected relatively powerful denoising methods based on a similar type of regressors (e.g., head movement, WM, CSF, and a global signal regressor) to explore the reproducibility of graph measures. Second, we used a publicly available dataset for which the imaging parameters and the protocol were already defined. As a result, we could not determine the effect of the sampling rate (repetition time) or the variations in protocol such as eyes open (with or without a fixation point) or eyes closed. Third, we looked at graph measures based on overall functional connectivity. In future studies, the reproducibility of graph measures for different brain states can be investigated.

## 5 CONCLUSIONS

We systematically investigated the reproducibility of graph measures of brain networks defined at the subject level as potential biomarkers in clinical work. The denoising pipeline did not affect the reproducibility. The reproducibility of graph measures of individual binary networks was insufficient especially when the density of the network was low. This was also the case for the reproducibility of nodal graph measures based on weighted or binary networks. For weighted networks, using the absolute value of the (partial) correlation as weight, was the method of choice and the reproducibility does not critically depend on the level of granularity.

## CONFLICTS OF INTEREST

The authors declare no competing financial interests.

## AUTHOR CONTRIBUTIONS

QR, RV, and PD designed the study; QR and PD performed the research; PD made the scripts used in this study; QR, TJ, JS, KM, RV, and PD wrote the paper and critically checked the content.

## Supporting information

Table S1‐S10

## Data Availability

The data that support the findings of this study are available from http://neuroinformatics.harvard.edu/gsp/.

## References

[brb31705-bib-0001] Arslan, S. , Ktena, S. I. , Makropoulos, A. , Robinson, E. C. , Rueckert, D. , & Parisot, S. (2018). Human brain mapping: A systematic comparison of parcellation methods for the human cerebral cortex. NeuroImage, 170, 5–30. 10.1016/j.neuroimage.2017.04.014 28412442

[brb31705-bib-0002] Bianciardi, M. , Fukunaga, M. , van Gelderen, P. , Horovitz, S. G. , de Zwart, J. A. , Shmueli, K. , & Duyn, J. H. (2009). Sources of functional magnetic resonance imaging signal fluctuations in the human brain at rest: A 7 T study. Magnetic Resonance Imaging, 27, 1019–1029. 10.1016/j.mri.2009.02.004 19375260 PMC3512098

[brb31705-bib-0003] Biswal, B. , Yetkin, F. Z. , Haughton, V. M. , & Hyde, J. S. (1995). Functional connectivity in the motor cortex of resting human brain using. Magnetic Resonance in Medicine, 34(9), 537–541. 10.1002/mrm.1910340409 8524021

[brb31705-bib-0004] Braun, U. , Plichta, M. M. , Esslinger, C. , Sauer, C. , Haddad, L. , Grimm, O. , … Meyer‐Lindenberg, A. (2012). Test‐retest reliability of resting‐state connectivity network characteristics using fMRI and graph theoretical measures. NeuroImage, 59(2), 1404–1412. 10.1016/j.neuroimage.2011.08.044 21888983

[brb31705-bib-0005] Bullmore, E. , & Sporns, O. (2009). Complex brain networks: Graph theoretical analysis of structural and functional systems. Nature Reviews Neuroscience, 10(3), 186–198. 10.1038/nrn2575 19190637

[brb31705-bib-0006] Burianová, H. , Faizo, N. L. , Gray, M. , Hocking, J. , Galloway, G. , & Reutens, D. (2017). Altered functional connectivity in mesial temporal lobe epilepsy. Epilepsy Research, 137, 45–52. 10.1016/j.eplepsyres.2017.09.001 28923408

[brb31705-bib-0007] Caballero‐Gaudes, C. , & Reynolds, R. C. (2017). Methods for cleaning the BOLD fMRI signal. NeuroImage, 154, 128–149. 10.1016/j.neuroimage.2016.12.018 27956209 PMC5466511

[brb31705-bib-0008] Chen, X. , Liao, X. , Dai, Z. , Lin, Q. , Wang, Z. , Li, K. , & He, Y. (2018). Topological analyses of functional connectomics: A crucial role of global signal removal, brain parcellation, and null models. Human Brain Mapping, 39, 4545–4564. 10.1002/hbm.24305 29999567 PMC6866637

[brb31705-bib-0009] Ciric, R. , Rosen, A. F. G. , Erus, G. , Cieslak, M. , Adebimpe, A. , Cook, P. A. , … Satterthwaite, T. D. (2018). Mitigating head motion artifact in functional connectivity MRI. Nature Protocols, 13, 2801–2826. 10.1038/s41596-018-0065-y 30446748 PMC8161527

[brb31705-bib-0010] Ciric, R. , Wolf, D. H. , Power, J. D. , Roalf, D. R. , Baum, G. L. , Ruparel, K. , … Satterthwaite, T. D. (2017). Benchmarking of participant‐level confound regression strategies for the control of motion artifact in studies of functional connectivity. NeuroImage, 154, 174–187. 10.1016/j.neuroimage.2017.03.020 28302591 PMC5483393

[brb31705-bib-0011] Dawson, D. A. , Lam, J. , Lewis, L. B. , Carbonell, F. , Mendola, J. D. , & Shmuel, A. (2016). Partial correlation‐based retinotopically organized resting‐state functional connectivity within and between areas of the visual cortex reflects more than cortical distance. Brain Connectivity, 6, 57–75. 10.1089/brain.2014.0331 26415043 PMC4744894

[brb31705-bib-0012] de Vos, F. , Koini, M. , Schouten, T. M. , Seiler, S. , van der Grond, J. , Lechner, A. , … Rombouts, S. A. R. B. (2018). A comprehensive analysis of resting state fMRI measures to classify individual patients with Alzheimer's disease. NeuroImage, 167, 62–72. 10.1016/j.neuroimage.2017.11.025 29155080

[brb31705-bib-0013] Desikan, R. S. , Ségonne, F. , Fischl, B. , Quinn, B. T. , Dickerson, B. C. , Blacker, D. , … Killiany, R. J. (2006). An automated labeling system for subdividing the human cerebral cortex on MRI scans into gyral based regions of interest. NeuroImage, 31, 968–980. 10.1016/j.neuroimage.2006.01.021 16530430

[brb31705-bib-0014] Dimitriadis, S. I. , Antonakakis, M. , Simos, P. G. , Fletcher, J. , & Papanicolaou, A. (2017). Data‐driven topological filtering based on orthogonal minimal spanning trees: Application to multi‐group MEG resting‐state connectivity. Brain Connectivity, 7, 661–670. 10.1089/brain.2017.0512 28891322 PMC6435350

[brb31705-bib-0015] Dimitriadis, S. I. , Drakesmith, M. , Bells, S. , Parker, G. D. , Linden, D. E. , & Jones, D. K. (2017). Improving the reliablity of network metrics in structural brain networks by integrating different network weighting strategies into a single graph. Front. Neurosci, 11, 694 10.3389/fnins.2017.00694 29311775 PMC5742099

[brb31705-bib-0016] Dimitriadis, S. I. , Salis, C. , Tarnanas, I. , & Linden, D. (2017). Topological filtering of dynamic functional brain networks unfolds informative chronnectomics: A novel data‐driven thresholding scheme based on orthogonal minimal spanning trees (OMSTs). Frontiers in Neuroinformatics, 11, 28 10.3389/fninf.2017.00028 28491032 PMC5405139

[brb31705-bib-0017] Du, H.‐X. , Liao, X.‐H. , Lin, Q.‐X. , Li, G.‐S. , Chi, Y.‐Z. , Liu, X. , … Xia, M.‐R. (2015). Test‐retest reliability of graph metrics in high‐resolution functional connectomics: A resting‐state functional MRI Study. CNS Neuroscience and Therapeutics, 21(10), 802–816. 10.1111/cns.12431 26212146 PMC6493187

[brb31705-bib-0018] Finn, J. D. (1974). A general model for multivariate analysis (p. 182). New York, NY: Holt, Rinehart & Winston.

[brb31705-bib-0019] Fox, M. D. , Snyder, A. Z. , Vincent, J. L. , & Raichle, M. E. (2007). Intrinsic fluctuations within cortical systems account for intertrial variability in human behavior. Neuron, 56, 171–184. 10.1016/j.neuron.2007.08.023 17920023

[brb31705-bib-0020] Fox, M. D. , Zhang, D. , Snyder, A. Z. , & Raichle, M. E. (2009). The global signal and observed anticorrelated resting state brain networks. Journal of Neurophysiology, 101(6), 3270–3283. 10.1152/jn.90777.2008 19339462 PMC2694109

[brb31705-bib-0021] Frazier, J. A. , Chiu, S. , Breeze, J. L. , Makris, N. , Lange, N. , Kennedy, D. N. , … Biederman, J. (2005). Structural brain magnetic resonance imaging of limbic and thalamic volumes in pediatric bipolar disorder. American Journal of Psychiatry, 162, 1256–1265. 10.1176/appi.ajp.162.7.1256 15994707

[brb31705-bib-0022] Friston, K. J. , Williams, S. , Howard, R. , Frackowiak, R. S. J. , & Turner, R. (1996). Movement‐related effects in fMRI time‐series. Magnetic Resonance in Medicine, 35, 346–355. 10.1002/mrm.1910350312 8699946

[brb31705-bib-0023] Fritz, H. C. J. , Ray, N. , Dyrba, M. , Sorg, C. , Teipel, S. , & Grothe, M. J. (2019). The corticotopic organization of the human basal forebrain as revealed by regionally selective functional connectivity profiles. Human Brain Mapping, 40, 868–878. 10.1002/hbm.24417 30311315 PMC6865372

[brb31705-bib-0024] Goldstein, J. M. , Seidman, L. J. , Makris, N. , Ahern, T. , O'Brien, L. M. , Caviness, V. S., Jr. , … Tsuang, M. T. (2007). Hypothalamic abnormalities in schizophrenia: Sex effects and genetic vulnerability. Biological Psychiatry, 15, 935–945. 10.1016/j.biopsych.2006.06.027 17046727

[brb31705-bib-0026] Gordon, E. M. , Laumann, T. O. , Gilmore, A. W. , Newbold, D. J. , Greene, D. J. , Berg, J. J. , … Dosenbach, N. U. F. (2017). Precision functional mapping of individual human brains. Neuron, 95(4), 791–807.e7. 10.1016/j.neuron.2017.07.011 28757305 PMC5576360

[brb31705-bib-0027] Greicius, M. D. , Krasnow, B. , Reiss, A. L. , & Menon, V. (2003). Functional connectivity in the resting brain: A network analysis of the default mode hypothesis. Proceedings of the National Academy of Sciences of the United States of America, 100, 253–258. 10.1073/pnas.0135058100 12506194 PMC140943

[brb31705-bib-0028] Greicius, M. D. , Srivastava, G. , Reiss, A. L. , & Menon, V. (2004). Default‐mode network activity distinguishes Alzheimer's disease from healthy aging: Evidence from functional MRI. Proceedings of the National Academy of Sciences, 101(13), 4637–4642. 10.1073/pnas.0308627101 PMC384799 15070770

[brb31705-bib-0029] Hallquist, M. N. , & Hillary, F. G. (2018). Graph theory approaches to functional network organization in brain disorders: A critique for a brave new small‐world. Network Neuroscience, 3, 1–26. 10.1162/netn_a_00054 30793071 PMC6326733

[brb31705-bib-0030] Holmes, A. J. , Hollinshead, M. O. , O'Keefe, T. M. , Petrov, V. I. , Fariello, G. R. , Wald, L. L. , … Buckner, R. L. (2015). Brain Genomics Superstruct Project initial data release with structural, functional, and behavioral measures. Scientific Data, 2, 150031 10.1038/sdata.2015.31 26175908 PMC4493828

[brb31705-bib-0031] Horn, A. , Wenzel, G. , Irmen, F. , Huebl, J. , Li, N. , Neumann, W.‐J. , … Kühn, A. A. (2019). Deep brain stimulation induced normalization of the human functional connectome in Parkinson's disease. Brain, 142, 3129–3143. 10.1093/brain/awz239 31412106

[brb31705-bib-0032] Hosseini, S. M. H. , Black, J. M. , Soriano, T. , Bugescu, N. , Martinez, R. , Raman, M. M. , … Hoeft, F. (2013). Topological properties of large‐scale structural brain networks in children with familial risk for reading difficulties. NeuroImage, 71, 260–274. 10.1016/j.neuroimage.2013.01.013 23333415 PMC3655726

[brb31705-bib-0033] Jackson, J. E. (1991). A user's guide to principal components. New York, NY: Wiley.

[brb31705-bib-0034] Johnson, K. A. , Sperling, R. A. , & Sepulcre, J. (2013). Functional connectivity in Alzheimer's disease: Measurement and meaning. Biological Psychiatry, 74, 318–319. 10.1016/j.biopsych.2013.07.010 23932342

[brb31705-bib-0035] Kabbara, A. , Khalil, M. , O'Neill, G. , Dujardin, K. , El Traboulsi, Y. , Wendling, F. , & Hassan, M. (2019). Detecting modular brain states in rest and task. Network Neuroscience, 3, 878–901. 10.1162/netn_a_00090 31410384 PMC6663471

[brb31705-bib-0036] Kaiser, M. , & Hilgetag, C. C. (2006). Nonoptimal component placement, but short processing paths, due to long‐distance projections in neural systems. PLoS Computational Biology, 2, e95 10.1371/journal.pcbi.0020095 16848638 PMC1513269

[brb31705-bib-0037] Kazeminejad, A. , & Sotero, R. C. (2019). The importance of anti‐correlations in graph theory based classification of autism spectrum disorder. BioRxiv. 1–16. 10.1101/557512 PMC7426475 32848533

[brb31705-bib-0038] Kirino, E. , Tanaka, S. , Fukuta, M. , Inami, R. , Inoue, R. , & Aoki, S. (2019). Functional connectivity of the caudate in Schizophrenia evaluated with simultaneous resting‐state functional MRI and electroencephalography recordings. Neuropsychobiology, 77, 165–175. 10.1159/000490429 30048962

[brb31705-bib-0039] Li, H. , Xue, Z. , Ellmore, T. M. , Frye, R. E. , & Wong, S. T. C. (2014). Network‐based analysis reveals stronger local diffusion‐based connectivity and different correlations with oral language skills in brains of children with high functioning autism spectrum disorders. Human Brain Mapping, 35, 396–413. 10.1002/hbm.22185 23008187 PMC6869619

[brb31705-bib-0040] Liang, X. , Wang, J. , Yan, C. , Shu, N. , Xu, K. , Gong, G. , & He, Y. (2012). Effects of different correlation metrics and preprocessing factors on small‐world brain functional networks: A resting‐state functional MRI study. PLoS One, 7, e32766 10.1371/journal.pone.0032766 22412922 PMC3295769

[brb31705-bib-0041] Lin, S. J. , Vavasour, I. , Kosaka, B. , Li, D. K. B. , Traboulsee, A. , MacKay, A. , & McKeown, M. J. (2018). Education, and the balance between dynamic and stationary functional connectivity jointly support executive functions in relapsing–remitting multiple sclerosis. Human Brain Mapping, 39, 5039–5049. 10.1002/hbm.24343 30240533 PMC6866468

[brb31705-bib-0042] Makris, N. , Goldstein, J. M. , Kennedy, D. , Hodge, S. M. , Caviness, V. S. , Faraone, S. V. , … Seidman, L. J. (2006). Decreased volume of left and total anterior insular lobule in schizophrenia. Schizophrenia Research, 83, 155–171. 10.1016/j.schres.2005.11.020 16448806

[brb31705-bib-0043] Marrelec, G. , Krainik, A. , Duffau, H. , Pélégrini‐Issac, M. , Lehéricy, S. , Doyon, J. , & Benali, H. (2006). Partial correlation for functional brain interactivity investigation in functional MRI. NeuroImage, 32, 228–237. 10.1016/j.neuroimage.2005.12.057 16777436

[brb31705-bib-0044] Murphy, K. , Birn, R. M. , Handwerker, D. A. , Jones, T. B. , & Bandettini, P. A. (2009). The impact of global signal regression on resting state correlations: Are anti‐correlated networks introduced? NeuroImage, 44(3), 893–905. 10.1016/j.neuroimage.2008.09.036 18976716 PMC2750906

[brb31705-bib-0045] Paldino, M. J. , Chu, Z. D. , Chapieski, M. L. , Golriz, F. , & Zhang, W. (2017). Repeatability of graph theoretical metrics derived from resting‐state functional networks in paediatric epilepsy patients. British Journal of Radiology, 90, 1074 10.1259/bjr.20160656 PMC5602170 28406312

[brb31705-bib-0046] Park, H.‐J. , & Friston, K. (2013). Structural and functional brain networks: From connections to cognition. Science, 342(6158), 1238411 10.1126/science.1238411 24179229

[brb31705-bib-0047] Parkes, L. , Fulcher, B. , Yücel, M. , & Fornito, A. (2018). An evaluation of the efficacy, reliability, and sensitivity of motion correction strategies for resting‐state functional MRI. NeuroImage, 171, 415–436. 10.1016/j.neuroimage.2017.12.073 29278773

[brb31705-bib-0048] Petrella, J. R. (2011). Use of graph theory to evaluate brain networks: A clinic tool for a small‐world? Radiology, 259, 317–320. 10.1148/radiol.11110380 21502388

[brb31705-bib-0049] Poldrack, R. A. , Laumann, T. O. , Koyejo, O. , Gregory, B. , Hover, A. , Chen, M.‐Y. , … Mumford, J. A. (2015). Long‐term neural and physiological phenotyping of a single human. Nature Communications, 6, 8885 10.1038/ncomms9885 PMC4682164 26648521

[brb31705-bib-0050] Power, J. D. , Barnes, K. A. , Snyder, A. Z. , Schlaggar, B. L. , & Petersen, S. E. (2012). Spurious but systematic correlations in functional connectivity MRI networks arise from subject motion. NeuroImage, 59, 2142–2154. 10.1016/j.neuroimage.2011.10.018 22019881 PMC3254728

[brb31705-bib-0051] Power, J. D. , Mitra, A. , Laumann, T. O. , Snyder, A. Z. , Schlaggar, B. L. , & Petersen, S. E. (2014). Methods to detect, characterize, and remove motion artifact in resting state fMRI. NeuroImage, 84, 320–341. 10.1016/j.neuroimage.2013.08.048 23994314 PMC3849338

[brb31705-bib-0052] Power, J. D. , Plitt, M. , Laumann, T. O. , & Martin, A. (2017). Sources and implications of whole‐brain fMRI signals in humans. NeuroImage, 146, 609–625. 10.1016/j.neuroimage.2016.09.038 27751941 PMC5321814

[brb31705-bib-0053] Rubinov, M. , & Sporns, O. (2010). Complex network measures of brain connectivity: Uses and interpretations. NeuroImage, 52(3), 1059–1069. 10.1016/j.neuroimage.2009.10.003 19819337

[brb31705-bib-0054] Satterthwaite, T. D. , Ciric, R. , Roalf, D. R. , Davatzikos, C. , Bassett, D. S. , & Wolf, D. H. (2019). Motion artifact in studies of functional connectivity: Characteristics and mitigation strategies. Human Brain Mapping, 40, 2033–2051. 10.1002/hbm.23665 29091315 PMC5930165

[brb31705-bib-0055] Satterthwaite, T. D. , Elliott, M. A. , Gerraty, R. T. , Ruparel, K. , Loughead, J. , Calkins, M. E. , … Wolf, D. H. (2013). An improved framework for confound regression and filtering for control of motion artifact in the preprocessing of resting‐state functional connectivity data. NeuroImage, 64(1), 240–256. 10.1016/j.neuroimage.2012.08.052 22926292 PMC3811142

[brb31705-bib-0056] Satterthwaite, T. D. , Wolf, D. H. , Loughead, J. , Ruparel, K. , Elliott, M. A. , Hakonarson, H. , … Gur, R. E. (2012). Impact of in‐scanner head motion on multiple measures of functional connectivity: Relevance for studies of neurodevelopment in youth. NeuroImage, 64, 240–256. 10.1016/j.neuroimage.2011.12.063 22233733 PMC3746318

[brb31705-bib-0057] Schwarz, A. J. , & McGonigle, J. (2011). Negative edges and soft thresholding in complex network analysis of resting state functional connectivity data. NeuroImage, 55(3), 1132–1146. 10.1016/j.neuroimage.2010.12.047 21194570

[brb31705-bib-0058] Shen, X. , Tokoglu, F. , Papademetris, X. , & Constable, R. T. (2013). Groupwise whole‐brain parcellation from resting‐state fMRI data for network node identification. NeuroImage, 82, 403–415. 10.1016/j.neuroimage.2013.05.081 23747961 PMC3759540

[brb31705-bib-0059] Shirer, W. R. , Jiang, H. , Price, C. M. , Ng, B. , & Greicius, M. D. (2015). Optimization of rs‐fMRI pre‐processing for enhanced signal‐noise separation, test‐retest reliability, and group discrimination. NeuroImage, 117, 67–79. 10.1016/j.neuroimage.2015.05.015 25987368

[brb31705-bib-0060] Smith, S. M. , Miller, K. L. , Salimi‐Khorshidi, G. , Webster, M. , Beckmann, C. F. , Nichols, T. E. , … Woolrich, M. W. (2011). Network modelling methods for FMRI. NeuroImage, 54, 875–891. 10.1016/j.neuroimage.2010.08.063 20817103

[brb31705-bib-0061] Smith, S. M. , Vidaurre, D. , Beckmann, C. F. , Glasser, M. F. , Jenkinson, M. , Miller, K. L. , … Van Essen, D. C. (2013). Functional connectomics from resting‐state fMRI. Trends in Cognitive Sciences, 17, 666–682. 10.1016/j.tics.2013.09.016 24238796 PMC4004765

[brb31705-bib-0062] Smyser, C. D. , Inder, T. E. , Shimony, J. S. , Hill, J. E. , Degnan, A. J. , Snyder, A. Z. , & Neil, J. J. (2010). Longitudinal analysis of neural network development in preterm infants. Cerebral Cortex, 20, 2852–2862. 10.1093/cercor/bhq035 20237243 PMC2978240

[brb31705-bib-0063] Sporns, O. (2011). The human connectome: A complex network. Annals of the New York Academy of Sciences, 1224, 109–125. 10.1111/j.1749-6632.2010.05888.x 21251014

[brb31705-bib-0064] Tzourio‐Mazoyer, N. , Landeau, B. , Papathanassiou, D. , Crivello, F. , Etard, O. , Delcroix, N. , … Joliot, M. (2002). Automated anatomical labeling of activations in SPM using a macroscopic anatomical parcellation of the MNI MRI single‐subject brain. NeuroImage, 15, 273–289. 10.1006/nimg.2001.0978 11771995

[brb31705-bib-0065] van den Heuvel, M. P. , Mandl, R. C. W. , Stam, C. J. , Kahn, R. S. , & Hulshoff Pol, H. E. (2010). Aberrant frontal and temporal complex network structure in Schizophrenia: A graph theoretical analysis. Journal of Neuroscience, 30, 15915–15926. 10.1523/JNEUROSCI.2874-10.2010 21106830 PMC6633761

[brb31705-bib-0066] van der Kouwe, A. J. W. , Benner, T. , Salat, D. H. , & Fischl, B. (2008). Brain morphometry with multiecho MPRAGE. NeuroImage, 40, 559–569. 10.1016/j.neuroimage.2007.12.025 18242102 PMC2408694

[brb31705-bib-0067] van Wijk, B. C. M. , Stam, C. J. , & Daffertshofer, A. (2010). Comparing brain networks of different size and connectivity density using graph theory. PLoS One, 5, e13701 10.1371/journal.pone.0013701 21060892 PMC2965659

[brb31705-bib-0068] Vandenberghe, R. , Wang, Y. , Nelissen, N. , Vandenbulcke, M. , Dhollander, T. , Sunaert, S. , & Dupont, P. (2013). The associative‐semantic network for words and pictures: Effective connectivity and graph analysis. Brain and Language, 127, 264–272. 10.1016/j.bandl.2012.09.005 23084460

[brb31705-bib-0069] Waheed, S. H. , Mirbagheri, S. , Agarwal, S. , Kamali, A. , Yahyavi‐Firouz‐Abadi, N. , Chaudhry, A. , … Sair, H. I. (2016). Reporting of resting‐state functional magnetic resonance imaging preprocessing methodologies. Brain Connect, 6, 663–668. 10.1089/brain.2016.0446 27507129

[brb31705-bib-0070] Wang, J. H. , Zuo, X. N. , Gohel, S. , Milham, M. P. , Biswal, B. B. , & He, Y. (2011). Graph theoretical analysis of functional brain networks: Test‐retest evaluation on short‐ and long‐term resting‐state functional MRI data. PLoS One, 6, e21976 10.1371/journal.pone.0021976 21818285 PMC3139595

[brb31705-bib-0071] Wang, Y. , Ghumare, E. , Vandenberghe, R. , & Dupont, P. (2017). Comparison of different generalizations of clustering coefficient and local efficiency for weighted undirected graphs. Neural Computation, 29, 313–331. 10.1162/NECO_a_00914 27870616

[brb31705-bib-0072] Wang, Y. K. , Kang, J. , Kemmer, P. B. , & Guo, Y. (2016). An efficient and reliable statistical method for estimating functional connectivity in large scale brain networks using partial correlation. Frontiers in Neuroscience, 10, 123 10.3389/fnins.2016.00123 27242395 PMC4876368

[brb31705-bib-0073] Wang, Y. U. , Nelissen, N. , Adamczuk, K. , De Weer, A.‐S. , Vandenbulcke, M. , Sunaert, S. , … Dupont, P. (2014). Reproducibility and robustness of graph measures of the associative‐semantic network. PLoS One, 9(12), e115215 10.1371/journal.pone.0115215 25500823 PMC4264875

[brb31705-bib-0074] Wobbrock, J. O. , Findlater, L. , Gergle, D. , & Higgins, J. J. (2011). The aligned rank transform for nonparametric factorial analyses using only anova procedures. ACM Digital Library, 143–146. 10.1145/1978942.1978963

[brb31705-bib-0075] Xiang, J. , Xue, J. , Guo, H. , Li, D. , Cui, X. , Niu, Y. , … Wang, B. (2019). Graph‐based network analysis of resting‐state fMRI: Test‐retest reliability of binarized and weighted networks. Brain Imaging and Behavior, 13. in press. 10.1007/s11682-019-00042-6 30734917

