# Supplementary material for: Reproducibility of graph measures at the subject level using resting‐state fMRI
Source: Brain Behav. 2020 Jul 2;10(8):e01705. doi: 10.1002/brb3.1705 (PMC7428495; doi:10.1002/brb3.1705)
Supplement: Supplementary file 1 — Table S1‐S10 [file BRB3-10-e01705-s001.docx]

# **Table S1:** Summary of global graph measures for binary networks

|  |  | **simple denoising strategy** | | |  |  |  |  |  |  |
| --- | --- | --- | --- | --- | --- | --- | --- | --- | --- | --- |
|  |  | correlations | |  |  | partial correlations | |  |  |  |
|  |  | values at time point 1 | | TRT (%) |  | values at time point 1 | | TRT (%) |  |  |
| **graph measure** | **density (%)** | mean | SD | mean | SD | mean | SD | mean |  | SD |
| λ_n_ | 5 | 3.732 | 0.396 | 13.7 | 10.9 | 3.221 | 0.391 | 14.7 |  | 14.3 |
|  | 10 | 2.794 | 0.162 | 7.8 | 6.2 | 2.614 | 0.206 | 9.4 |  | 9.1 |
|  | 20 | 2.031 | 0.085 | 5.6 | 5.1 | 2.024 | 0.123 | 7.4 |  | 6.4 |
|  | 30 | 1.727 | 0.042 | 2.8 | 3.4 | 1.750 | 0.072 | 5.0 |  | 5.7 |
|  | 40 | 1.588 | 0.011 | 0.5 | 0.9 | 1.599 | 0.031 | 2.6 |  | 4.2 |
| C_n_ | 5 | 0.340 | 0.041 | 14.1 | 10.1 | 0.289 | 0.031 | 11.4 |  | 9.8 |
|  | 10 | 0.435 | 0.031 | 8.2 | 5.8 | 0.403 | 0.038 | 10.5 |  | 9.2 |
|  | 20 | 0.505 | 0.036 | 9.0 | 7.0 | 0.492 | 0.052 | 11.1 |  | 9.9 |
|  | 30 | 0.534 | 0.038 | 9.1 | 7.5 | 0.535 | 0.062 | 13.2 |  | 10.8 |
|  | 40 | 0.561 | 0.038 | 8.3 | 7.0 | 0.568 | 0.060 | 12.5 |  | 9.7 |
| E_n_ | 5 | 0.229 | 0.034 | 20.5 | 15.0 | 0.217 | 0.048 | 27.3 |  | 21.4 |
|  | 10 | 0.396 | 0.032 | 10.6 | 9.7 | 0.371 | 0.061 | 21.6 |  | 18.0 |
|  | 20 | 0.556 | 0.019 | 4.1 | 4.1 | 0.538 | 0.053 | 11.3 |  | 13.8 |
|  | 30 | 0.642 | 0.009 | 1.5 | 1.8 | 0.631 | 0.033 | 5.9 |  | 9.8 |
|  | 40 | 0.699 | 0.002 | 0.3 | 0.5 | 0.694 | 0.021 | 3.0 |  | 6.7 |
| BC_n_ | 5 | 0.021 | 0.006 | 40.9 | 31.6 | 0.015 | 0.006 | 53.7 |  | 41.1 |
|  | 10 | 0.018 | 0.002 | 12.3 | 14.3 | 0.015 | 0.003 | 28.5 |  | 34.0 |
|  | 20 | 0.011 | 0.001 | 10.1 | 8.9 | 0.011 | 0.001 | 16.5 |  | 20.4 |
|  | 30 | 0.008 | 0.000 | 6.3 | 7.5 | 0.008 | 0.001 | 11.8 |  | 15.9 |
|  | 40 | 0.007 | 0.000 | 1.5 | 2.4 | 0.007 | 0.000 | 7.5 |  | 12.6 |
|  |  |  |  |  |  |  |  |  |  |  |
|  |  | **complex denoising strategy** | | |  |  |  |  |  |  |
|  |  | correlations | |  |  | partial correlations | |  |  |  |
|  |  | values at time point 1 | | TRT (%) |  | values at time point 1 | | TRT (%) |  |  |
| **graph measure** | **density (%)** | mean | SD | mean | SD | mean | SD | mean |  | SD |
| λ_n_ | 5 | 3.765 | 0.453 | 13.0 | 10.4 | 3.139 | 0.424 | 14.7 |  | 13.0 |
|  | 10 | 2.782 | 0.150 | 7.3 | 6.2 | 2.621 | 0.206 | 10.2 |  | 10.2 |
|  | 20 | 2.030 | 0.086 | 5.3 | 5.2 | 2.059 | 0.146 | 7.7 |  | 7.4 |
|  | 30 | 1.726 | 0.038 | 2.7 | 2.9 | 1.759 | 0.114 | 5.3 |  | 6.8 |
|  | 40 | 1.587 | 0.007 | 0.5 | 0.9 | 1.601 | 0.084 | 3.2 |  | 5.6 |
| C_n_ | 5 | 0.342 | 0.029 | 11.7 | 8.5 | 0.294 | 0.035 | 12.6 |  | 8.4 |
|  | 10 | 0.441 | 0.029 | 7.3 | 5.5 | 0.408 | 0.040 | 10.2 |  | 7.8 |
|  | 20 | 0.506 | 0.033 | 8.5 | 7.0 | 0.500 | 0.050 | 10.3 |  | 9.0 |
|  | 30 | 0.535 | 0.037 | 9.1 | 7.1 | 0.545 | 0.060 | 11.0 |  | 9.6 |
|  | 40 | 0.561 | 0.035 | 8.0 | 6.7 | 0.576 | 0.060 | 9.9 |  | 9.6 |
| E_n_ | 5 | 0.229 | 0.035 | 20.8 | 14.8 | 0.208 | 0.048 | 26.6 |  | 20.5 |
|  | 10 | 0.397 | 0.032 | 10.3 | 9.4 | 0.363 | 0.064 | 18.8 |  | 17.0 |
|  | 20 | 0.556 | 0.017 | 3.9 | 3.8 | 0.535 | 0.052 | 10.4 |  | 13.0 |
|  | 30 | 0.642 | 0.007 | 1.4 | 1.7 | 0.627 | 0.041 | 5.7 |  | 9.3 |
|  | 40 | 0.699 | 0.001 | 0.3 | 0.8 | 0.691 | 0.031 | 3.2 |  | 7.2 |
| BC_n_ | 5 | 0.021 | 0.007 | 39.3 | 31.7 | 0.014 | 0.006 | 50.2 |  | 41.4 |
|  | 10 | 0.018 | 0.002 | 12.8 | 12.1 | 0.015 | 0.004 | 30.6 |  | 32.0 |
|  | 20 | 0.011 | 0.001 | 9.9 | 8.3 | 0.011 | 0.002 | 16.5 |  | 22.2 |
|  | 30 | 0.008 | 0.000 | 5.9 | 6.4 | 0.008 | 0.001 | 12.8 |  | 18.9 |
|  | 40 | 0.007 | 0.000 | 1.4 | 2.2 | 0.007 | 0.001 | 8.9 |  | 19.9 |

# **Table S2:** Summary of normalized global graph measures for weighted networks (analysis 1b)

| **A.** raw (partial) correlation as weight | simple denoising strategy | | |  |  |  |  |  | complex denoising strategy | | | |  |  |  |  |
| --- | --- | --- | --- | --- | --- | --- | --- | --- | --- | --- | --- | --- | --- | --- | --- | --- |
|  | correlations | |  |  | partial correlations | | |  | correlations | |  |  | partial correlations | | |  |
|  | values at time point 1 | | TRT (%) |  | values at time point 1 | | TRT (%) |  | values at time point 1 | | TRT (%) |  | values at time point 1 | | TRT (%) |  |
| **Normalized graph measure** | mean | SD | mean | SD | mean | SD | mean | SD | mean | SD | mean | SD | mean | SD | mean | SD |
| λ_n_ | 1.121 | 0.017 | 2.0 | 1.7 | 1.125 | 0.030 | 2.7 | 2.0 | 1.122 | 0.017 | 2.0 | 1.5 | 1.127 | 0.031 | 2.9 | 2.6 |
| C_n_ | 1.032 | 0.009 | 1.1 | 0.9 | 1.036 | 0.013 | 1.4 | 1.0 | 1.031 | 0.008 | 1.1 | 0.8 | 1.038 | 0.013 | 1.3 | 1.1 |
| E_n_ | 0.929 | 0.007 | 1.0 | 0.8 | 0.930 | 0.012 | 1.6 | 1.2 | 0.929 | 0.007 | 1.0 | 0.8 | 0.929 | 0.015 | 1.7 | 1.5 |
| BC_n_ | 0.921 | 0.023 | 3.3 | 2.8 | 0.916 | 0.047 | 6.2 | 5.4 | 0.921 | 0.023 | 3.3 | 3.0 | 0.907 | 0.059 | 6.0 | 6.8 |
|  |  |  |  |  |  |  |  |  |  |  |  |  |  |  |  |  |
| **B.** weights based on transformed Z values | simple denoising strategy | | |  |  |  |  |  | complex denoising strategy | | | |  |  |  |  |
|  | correlations | |  |  | partial correlations | | |  | correlations | |  |  | partial correlations | | |  |
|  | values at time point 1 | | TRT (%) |  | values at time point 1 | | TRT (%) |  | values at time point 1 | | TRT (%) |  | values at time point 1 | | TRT (%) |  |
| **Normalized graph measure** | mean | SD | mean | SD | mean | SD | mean | SD | mean | SD | mean | SD | mean | SD | mean | SD |
| λ_n_ | 1.000 | 0.000 | 0.0 | 0.0 | 1.077 | 0.219 | 4.4 | 7.7 | 1.000 | 0.000 | 0.0 | 0.0 | 1.067 | 0.176 | 3.9 | 6.9 |
| C_n_ | 1.008 | 0.002 | 0.3 | 0.3 | 1.113 | 0.067 | 3.3 | 3.4 | 1.008 | 0.002 | 0.3 | 0.2 | 1.111 | 0.077 | 3.6 | 4.4 |
| E_n_ | 1.000 | 0.000 | 0.0 | 0.0 | 0.969 | 0.044 | 1.9 | 2.9 | 1.000 | 0.000 | 0.0 | 0.0 | 0.972 | 0.044 | 2.0 | 3.5 |
| BC_n_ | 1.000 | 0.000 | 0.0 | 0.0 | 1.012 | 0.080 | 2.5 | 5.6 | 1.000 | 0.000 | 0.0 | 0.0 | 1.008 | 0.070 | 2.4 | 5.6 |

# **Table S3:** Summary of normalized nodal graph measures for weighted networks

|  | simple denoising strategy | | | |  |  |  |  |
| --- | --- | --- | --- | --- | --- | --- | --- | --- |
| **A.** raw (partial) correlation as weight | correlations | |  |  | partial correlations | | |  |
|  | values at time point 1 | | TRT (%) |  | values at time point 1 | | TRT (%) |  |
| **Normalized graph measure** | Mean  (range) | SD | Mean  (range) | SD | Mean  (range) | SD | Mean  (range) | SD |
| nodal average path length | 1.033 – 1.212 | 0.075 – 0.157 | 8.2 – 14.5 | 6.5 – 11.1 | 1.079 – 1.157 | 0.093 – 0.194 | 10.8 – 16.6 | 8.1 – 14.1 |
| local clustering coefficient | 1.016 – 1.048 | 0.020 – 0.035 | 2.1 – 3.9 | 1.6 – 2.8 | 1.027 – 1.046 | 0.026 – 0.043 | 2.7 – 4.5 | 2.1 – 3.5 |
| nodal efficiency | 0.836 – 1.341 | 0.279 – 0.383 | 27.7 – 46.2 | 20.4 – 33.8 | 0.983 – 1.196 | 0.319 – 0.484 | 35.1 – 51.4 | 26.1 – 39.9 |
| local betweenness centrality | 0.533 – 1.388 | 0.448 – 0.914 | 63.8 – 99.0 | 43.9 – 64.8 | 0.743 – 1.189 | 0.565 – 0.978 | 85.0 – 117.6 | 55.2 – 68.8 |
|  |  |  |  |  |  |  |  |  |
|  | complex denoising strategy | | | |  |  |  |  |
| **B.** raw (partial) correlation as weight | correlations | |  |  | partial correlations | | |  |
|  | values at time point 1 | | TRT (%) |  | values at time point 1 | | TRT (%) |  |
| **Normalized graph measure** | Mean  (range) | SD | Mean  (range) | SD | Mean  (range) | SD  (range) | Mean  (range) | SD |
| nodal average path length | 1.046 – 1.218 | 0.085 – 0.152 | 8.1 – 15.1 | 6.1 – 11.1 | 1.085 – 1.174 | 0.109 – 0.234 | 11.2 – 17.8 | 8.5 – 14.4 |
| local clustering coefficient | 1.017 – 1.053 | 0.018 – 0.036 | 2.3 – 4.1 | 1.5 – 3.1 | 1.029 – 1.046 | 0.026 – 0.042 | 2.8 – 4.4 | 2.1 -3.4 |
| nodal efficiency | 0.848 – 1.319 | 0.243 – 0.390 | 26.1 – 50.6 | 20.4 – 34.8 | 0.949 – 1.204 | 0.322 – 0.487 | 36.0 – 52.7 | 23.9 – 40.5 |
| local betweenness centrality | 0.516 – 1.417 | 0.449 – 0.965 | 65.8 – 113.0 | 43.9 – 65.8 | 0.631 – 1.144 | 0.567 – 1.615 | 84.7– 116.2 | 50.4 – 72.3 |

|  | simple denoising strategy | | | |  |  |  |  |
| --- | --- | --- | --- | --- | --- | --- | --- | --- |
| **C.** weights based on transformed Z values | correlations | |  |  | partial correlations | | |  |
|  | values at time point 1 | | TRT (%) |  | values at time point 1 | | TRT (%) |  |
| **Normalized graph measure** | Mean  (range) | SD | Mean  (range) | SD | Mean  (range) | SD | Mean  (range) | SD |
| nodal average path length | 0.961 – 1.037 | 0.048 – 0.085 | 5.1 – 8.8 | 3.9 – 6.4 | 1.028 – 1.255 | 0.131 – 1.736 | 10.2– 16.6 | 8.4 – 21.8 |
| local clustering coefficient | 1.005 – 1.012 | 0.008 – 0.013 | 0.9 – 1.4 | 0.6 – 1.1 | 1.097 – 1.127 | 0.061 – 0.112 | 5.4 – 7.9 | 4.0 – 7.3 |
| nodal efficiency | 0.977 – 1.043 | 0.040 – 0.075 | 4.0– 7.9 | 3.1 – 8.9 | 1.028 – 1.161 | 0.188 – 0.344 | 21.5 – 37.4 | 20.3 – 39.4 |
| local betweenness centrality | 0.529 – 1.464 | 0.499 – 1.165 | 70.1 – 110.8 | 49.2 – 71.0 | 0.839 – 1.382 | 0.585 – 1.230 | 83.8 – 115.1 | 51.7 – 67.9 |
|  |  |  |  |  |  |  |  |  |
|  | complex denoising strategy | | | |  |  |  |  |
| **D.** weights based on transformed Z values | correlations | |  |  | partial correlations | | |  |
|  | values at time point 1 | | TRT (%) |  | values at time point 1 | | TRT (%) |  |
| **Normalized graph measure** | Mean  (range) | SD | Mean  (range) | SD | Mean  (range) | SD | Mean  (range) | SD |
| nodal average path length | 0.963 – 1.044 | 0.047 – 0.077 | 4.8 – 8.9 | 3.6 – 6.6 | 1.020 – 1.149 | 0.115 – 0.660 | 10.7 – 17.3 | 8.1 – 20.5 |
| local clustering coefficient | 1.005 – 1.012 | 0.008 – 0.012 | 0.9– 1.3 | 0.7 – 1.0 | 1.087 – 1.131 | 0.055 – 0.137 | 5.3 – 8.1 | 4.0 – 8.9 |
| nodal efficiency | 0.973 – 1.037 | 0.038 – 0.073 | 4.0 – 8.5 | 3.1 – 7.4 | 0.989 – 1.166 | 0.219 – 0.462 | 22.0 – 38.3 | 18.7 – 42.1 |
| local betweenness centrality | 0.516 – 1.440 | 0.442 – 1.298 | 72.8– 126.1 | 51.6 – 70.8 | 0.704 – 1.246 | 0.562 – 1.923 | 80.6 – 119.7 | 52.4 – 70.9 |

Note: the mean and SD represent the mean and SD of the nodal graph measure or TRT across subjects.

# **Table S4:** Normalized global graph measures for weighted networks with different strategies to handle negative correlation values (analysis2)

| **A. Correlations** | abs |  |  |  | pos |  |  |  | neg |  |  |  |
| --- | --- | --- | --- | --- | --- | --- | --- | --- | --- | --- | --- | --- |
|  | values at time point 1 | | TRT (%) |  | values at time point 1 | | TRT (%) |  | values at time  point 1 | | TRT (%) |  |
| Normalized graph measure | mean | SD | mean | SD | mean | SD | mean | SD | mean | SD | mean | SD |
| λ_n_ | 1.121 | 0.017 | 2.0 | 1.7 | 1.367 | 0.068 | 6.2 | 5.4 | 1.075 | 0.029 | 3.0 | 2.7 |
| C_n_ | 1.032 | 0.009 | 1.1 | 0.9 | 1.618 | 0.090 | 6.2 | 5.2 | 0.508 | 0.069 | 16.9 | 13.4 |
| E_n_ | 0.929 | 0.007 | 1.0 | 0.8 | 0.816 | 0.022 | 3.3 | 2.8 | 0.957 | 0.013 | 1.7 | 1.4 |
| BC_n_ | 0.921 | 0.023 | 3.3 | 2.8 | 1.318 | 0.066 | 5.6 | 4.5 | 0.997 | 0.018 | 1.9 | 1.4 |
|  |  |  |  |  |  |  |  |  |  |  |  |  |
| **B. Partial correlations** | abs |  |  |  | pos |  |  |  | neg |  |  |  |
|  | values at time point 1 | | TRT (%) |  | values at time point 1 | | TRT (%) |  | values at time  point 1 | | TRT (%) |  |
| Normalized graph measure | mean | SD | mean | SD | mean | SD | mean | SD | mean | SD | mean | SD |
| λ_n_ | 1.125 | 0.030 | 2.7 | 2.0 | 1.082 | 0.040 | 4.0 | 2.8 | 1.362 | 0.147 | 12.3 | 11.1 |
| C_n_ | 1.036 | 0.013 | 1.4 | 1.0 | 0.473 | 0.102 | 33.0 | 35.4 | 1.601 | 0.128 | 9.7 | 7.7 |
| E_n_ | 0.930 | 0.012 | 1.6 | 1.2 | 0.955 | 0.020 | 2.2 | 1.5 | 0.826 | 0.037 | 5.5 | 4.1 |
| BC_n_ | 0.916 | 0.047 | 6.2 | 5.4 | 0.997 | 0.018 | 1.9 | 1.4 | 1.331 | 0.124 | 10.9 | 11.2 |

|  |  | Main Effects | | | | |  | Interactions | |
| --- | --- | --- | --- | --- | --- | --- | --- | --- | --- |
| **C. Statistical comparison** |  | handling negative values | |  | Correlation | |  | handling negative values | |
|  |  | (abs, pos, neg) | |  | (Cor vs Parco) | |  | * | |
|  |  |  | |  |  | |  | Correlation | |
| Normalized graph measure | df | F | *p* | df | F | *p* | df | F | *p* |
| λ_n_ | (2,340) | 56.0 | **< 1.0E-15** | (1,340) | 66.5 | **6.9E-15** | (2,340) | 59.4 | **< 1.0E-15** |
| C_n_ | (2,340) | 168.4 | **< 1.0E-15** | (1,340) | 71.5 | **< 1.0E-15** | (2,340) | 91.1 | **< 1.0E-15** |
| E_n_ | (2,340) | 50.3 | **< 1.0E-15** | (1,340) | 46.1 | **5.0E-11** | (2,340) | 41.6 | **< 1.0E-15** |
| BC_n_ | (2,340) | 11.9 | **9.9E-06** | (1,340) | 41.1 | **4.9E-10** | (2,340) | 79.9 | **< 1.0E-15** |

Note: P-values are uncorrected but those in bold are significant after a Bonferroni correction (p<0.05) correcting for 4 tests.

# **Table S5:** Summary of global graph measures for **binary networks** for the Shen50 atlas after applying a data-driven topological filtering approach based on orthogonal minimal spanning trees (Dimitriadis et al. 2017a,b)

| raw (partial) correlation as weight | simple denoising strategy | | |  |  |  |  |  | complex denoising strategy | | | |  |  |  |  |
| --- | --- | --- | --- | --- | --- | --- | --- | --- | --- | --- | --- | --- | --- | --- | --- | --- |
|  | correlations | |  |  | partial correlations | | |  | correlations | |  |  | partial correlations | | |  |
|  | values at time point 1 | | TRT (%) |  | values at time point 1 | | TRT (%) |  | values at time point 1 | | TRT (%) |  | values at time point 1 | | TRT (%) |  |
| **Normalized graph measure** | mean | SD | mean | SD | mean | SD | mean | SD | mean | SD | mean | SD | mean | SD | mean | SD |
| λ_n_ | 1.171 | 0.035 | 3.0 | 2.4 | 1.133 | 0.069 | 6.0 | 7.0 | 1.216 | 0.035 | 3.2 | 2.5 | 1.136 | 0.069 | 6.0 | 6.7 |
| C_n_ | 5.420 | 0.511 | 10.2 | 8.0 | 4.846 | 0.691 | 17.6 | 11.1 | 5.731 | 0.560 | 10.0 | 7.8 | 4.862 | 0.673 | 15.7 | 13.9 |
| E_n_ | 0.895 | 0.017 | 2.0 | 1.6 | 0.919 | 0.032 | 3.9 | 4.3 | 0.872 | 0.017 | 2.1 | 1.7 | 0.918 | 0.034 | 4.0 | 4.2 |
| BC_n_ | 1.276 | 0.055 | 3.5 | 3.5 | 1.217 | 0.112 | 8.9 | 10.2 | 1.287 | 0.050 | 4.5 | 3.2 | 1.223 | 0.116 | 8.7 | 9.8 |

| Main Effects |  | Denoising | | Correlation | | Interaction | |
| --- | --- | --- | --- | --- | --- | --- | --- |
|  |  | (S vs C) | | (Cor vs Parco) | |  | |
|  | df | F | *p* | F | *p* | F | *p* |
| λ_n_ | (1,204) | 0.08 | 0.78 | 7.09 | **8.34E-03** | 5.25E-05 | 0.99 |
| C_n_ | (1,204) | 2.08 | 0.15 | 24.23 | **1.76E-06** | 2.11 | 0.15 |
| E_n_ | (1,204) | 0.36 | 0.55 | 10.74 | **1.23E-03** | 0.11 | 0.74 |
| BC_n_ | (1,204) | 0.12 | 0.89 | 7.88 | **5.47E-03** | 0.06 | 0.81 |

Note: P-values are uncorrected but those in bold are significant after a Bonferroni correction (p<0.05) correcting for 4 tests.

**Table S6:** Summary of normalized global graph measures for **weighted networks** for the Shen50 atlas after applying a data-driven topological filtering approach based on orthogonal minimal spanning trees (Dimitriadis et al. 2017a,b)

| raw (partial) correlation as weight | simple denoising strategy | | |  |  |  |  |  | complex denoising strategy | | | |  |  |  |  |
| --- | --- | --- | --- | --- | --- | --- | --- | --- | --- | --- | --- | --- | --- | --- | --- | --- |
|  | correlations | |  |  | partial correlations | | |  | correlations | |  |  | partial correlations | | |  |
|  | values at time point 1 | | TRT (%) |  | values at time point 1 | | TRT (%) |  | values at time point 1 | | TRT (%) |  | values at time point 1 | | TRT (%) |  |
| **Normalized graph measure** | mean | SD | mean | SD | mean | SD | mean | SD | mean | SD | mean | SD | mean | SD | mean | SD |
| λ_n_ | 1.207 | 0.040 | 3.3 | 2.5 | 1.163 | 0.074 | 6.5 | 6.8 | 1.216 | 0.035 | 3.2 | 2.5 | 1.168 | 0.076 | 6.3 | 6.8 |
| C_n_ | 5.590 | 0.543 | 10.5 | 7.2 | 5.004 | 0.678 | 15.2 | 11.1 | 5.691 | 0.526 | 10.6 | 7.2 | 5.052 | 0.574 | 14.3 | 12.3 |
| E_n_ | 0.876 | 0.019 | 2.3 | 1.7 | 0.906 | 0.034 | 4.2 | 4.2 | 0.871 | 0.018 | 2.1 | 1.8 | 0.904 | 0.036 | 4.1 | 4.3 |
| BC_n_ | 1.272 | 0.056 | 4.4 | 3.5 | 1.213 | 0.113 | 10.3 | 10.3 | 1.286 | 0.049 | 4.5 | 3.2 | 1.220 | 0.118 | 8.8 | 9.9 |

| Main Effects |  | Denoising | | Correlation | | Interaction | |
| --- | --- | --- | --- | --- | --- | --- | --- |
|  |  | (S vs C) | | (Cor vs Parco) | |  | |
|  | df | F | *p* | F | *p* | F | *p* |
| λ_n_ | (1,204) | 0.27 | 0.60 | 12.08 | **6.24E-04** | 0.04 | 0.84 |
| C_n_ | (1,204) | 0.51 | 0.47 | 6.27 | 0.01 | 0.65 | 0.42 |
| E_n_ | (1,204) | 0.08 | 0.77 | 11.69 | **7.57E-04** | 0.25 | 0.62 |
| BC_n_ | (1,204) | 2.17E-04 | 0.99 | 8.83 | **3.33E-03** | 0.16 | 0.69 |

Note: P-values are uncorrected but those in bold are significant after a Bonferroni correction (p<0.05) correcting for 4 tests.

# **Table S7:** Summary of global graph measures for binary networks for the Oxford-Harvard atlas

|  |  | **simple denoising strategy** | | |  |  |  |  |  |  |
| --- | --- | --- | --- | --- | --- | --- | --- | --- | --- | --- |
|  |  | correlations | |  |  | partial correlations | |  |  |  |
|  |  | values at time point 1 | | TRT (%) |  | values at time point 1 | | TRT (%) |  |  |
| **graph measure** | **density (%)** | mean | SD | mean | SD | mean | SD | mean |  | SD |
| λ_n_ | 5 | 0.789 | 0.220 | 33.7 | 21.7 | 0.957 | 0.143 | 16.7 |  | 13.6 |
|  | 10 | 1.179 | 0.152 | 15.1 | 13.6 | 1.059 | 0.043 | 4.0 |  | 3.4 |
|  | 20 | 1.187 | 0.077 | 6.9 | 5.8 | 1.043 | 0.022 | 1.9 |  | 1.6 |
|  | 30 | 1.078 | 0.042 | 4.6 | 3.4 | 1.012 | 0.010 | 0.7 |  | 0.6 |
|  | 40 | 1.018 | 0.020 | 2.3 | 2.6 | 1.001 | 0.001 | 0.1 |  | 0.1 |
| C_n_ | 5 | 8.686 | 2.218 | 28.8 | 19.9 | 4.472 | 1.676 | 44.8 |  | 36.5 |
|  | 10 | 4.424 | 0.623 | 15.9 | 11.9 | 2.659 | 0.498 | 18.5 |  | 15.3 |
|  | 20 | 2.591 | 0.194 | 9.5 | 6.2 | 1.706 | 0.197 | 10.5 |  | 7.3 |
|  | 30 | 1.871 | 0.145 | 9.1 | 6.2 | 1.323 | 0.111 | 6.6 |  | 5.2 |
|  | 40 | 1.469 | 0.111 | 10.2 | 6.6 | 1.155 | 0.058 | 4.2 |  | 3.1 |
| E_n_ | 5 | 0.520 | 0.125 | 28.1 | 18.4 | 0.812 | 0.117 | 15.3 |  | 12.8 |
|  | 10 | 0.690 | 0.103 | 20.0 | 15.8 | 0.914 | 0.058 | 5.7 |  | 4.6 |
|  | 20 | 0.881 | 0.053 | 8.0 | 6.1 | 0.975 | 0.014 | 1.3 |  | 1.2 |
|  | 30 | 0.960 | 0.028 | 3.5 | 4.3 | 0.995 | 0.004 | 0.3 |  | 0.3 |
|  | 40 | 0.992 | 0.011 | 1.1 | 1.8 | 1.000 | 0.000 | 0.0 |  | 0.1 |
| BC_n_ | 5 | 0.352 | 0.279 | 83.9 | 46.7 | 0.750 | 0.264 | 42.1 |  | 33.9 |
|  | 10 | 0.977 | 0.328 | 42.6 | 38.1 | 1.031 | 0.103 | 9.6 |  | 10.3 |
|  | 20 | 1.334 | 0.164 | 14.7 | 11.8 | 1.088 | 0.049 | 4.1 |  | 3.6 |
|  | 30 | 1.179 | 0.100 | 9.7 | 9.5 | 1.028 | 0.023 | 1.7 |  | 1.4 |
|  | 40 | 1.048 | 0.051 | 5.4 | 5.7 | 1.003 | 0.003 | 0.3 |  | 0.4 |
|  |  |  |  |  |  |  |  |  |  |  |
|  |  | **complex denoising strategy** | | |  |  |  |  |  |  |
|  |  | correlations | |  |  | partial correlations | |  |  |  |
|  |  | values at time point 1 | | TRT (%) |  | values at time point 1 | | TRT (%) |  |  |
| **graph measure** | **density (%)** | mean | SD | mean | SD | mean | SD | mean |  | SD |
| λ_n_ | 5 | 0.762 | 0.241 | 32.2 | 23.4 | 0.956 | 0.126 | 17.8 |  | 13.4 |
|  | 10 | 1.190 | 0.178 | 16.3 | 16.5 | 1.061 | 0.046 | 4.9 |  | 3.6 |
|  | 20 | 1.181 | 0.087 | 7.3 | 5.7 | 1.042 | 0.023 | 1.8 |  | 1.8 |
|  | 30 | 1.078 | 0.042 | 4.5 | 3.3 | 1.012 | 0.008 | 0.7 |  | 0.6 |
|  | 40 | 1.018 | 0.020 | 1.8 | 2.0 | 1.001 | 0.001 | 0.1 |  | 0.2 |
| C_n_ | 5 | 8.633 | 2.470 | 27.5 | 19.6 | 4.540 | 1.831 | 43.9 |  | 31.8 |
|  | 10 | 4.377 | 0.574 | 14.4 | 10.5 | 2.574 | 0.471 | 20.6 |  | 14.8 |
|  | 20 | 2.563 | 0.187 | 9.0 | 7.4 | 1.702 | 0.183 | 9.6 |  | 7.6 |
|  | 30 | 1.863 | 0.153 | 9.7 | 7.3 | 1.327 | 0.105 | 6.3 |  | 5.3 |
|  | 40 | 1.467 | 0.107 | 9.1 | 6.7 | 1.155 | 0.053 | 4.2 |  | 3.4 |
| E_n_ | 5 | 0.501 | 0.120 | 27.7 | 15.9 | 0.810 | 0.099 | 15.2 |  | 13.2 |
|  | 10 | 0.687 | 0.114 | 19.0 | 17.1 | 0.916 | 0.053 | 5.8 |  | 5.4 |
|  | 20 | 0.883 | 0.062 | 7.4 | 7.5 | 0.974 | 0.016 | 1.3 |  | 1.5 |
|  | 30 | 0.960 | 0.028 | 3.0 | 3.1 | 0.994 | 0.004 | 0.4 |  | 0.5 |
|  | 40 | 0.993 | 0.008 | 1.0 | 1.8 | 0.999 | 0.001 | 0.1 |  | 0.1 |
| BC_n_ | 5 | 0.326 | 0.272 | 80.7 | 45.5 | 0.735 | 0.241 | 44.9 |  | 34.2 |
|  | 10 | 1.007 | 0.386 | 46.7 | 39.4 | 1.038 | 0.107 | 13.1 |  | 11.1 |
|  | 20 | 1.329 | 0.208 | 15.6 | 15.0 | 1.084 | 0.051 | 4.0 |  | 3.9 |
|  | 30 | 1.178 | 0.097 | 9.1 | 6.3 | 1.030 | 0.020 | 1.7 |  | 1.4 |
|  | 40 | 1.047 | 0.053 | 4.5 | 4.5 | 1.003 | 0.004 | 0.4 |  | 0.4 |

# **Table S8:** Comparison of denoising strategy and type of correlation on test-retest values for global graph measures of binary networks for the Oxford-Harvard atlas

|  |  |  | **Main Effects** | | | | **Interaction** | |
| --- | --- | --- | --- | --- | --- | --- | --- | --- |
|  |  |  | **Denoising**  (Simple vs Complex) | | **Correlation**  (Pearson vs partial correlation) | | **Denoising**  *****  **Correlation** | |
|  |  |  |  |  |  |  |  |  |
|  |  |  |  |  |  |  |  |  |
| Graph measure | Density (%) | df | F | *p* | F | *p* | F | *p* |
| λ_n_ | 5 | (1,204) | 0.02 | 0.90 | 49.99 | **2.41E-11** | 0.54 | 0.46 |
|  | 10 | (1,204) | 0.58 | 0.45 | 83.75 | **<2.00E-16** | 0.01 | 0.91 |
|  | 20 | (1,204) | 0.19 | 0.67 | 155.68 | **<2.00E-16** | 0.67 | 0.41 |
|  | 30 | (1,204) | 0.38 | 0.54 | 414.68 | **<2.00E-16** | 0.31 | 0.58 |
|  | 40 | (1,204) | 13.08 | **3.76E-04** | 337.02 | **<2.22E-16** | 20.12 | **1.22E-05** |
| C_n_ | 5 | (1,204) | 0.06 | 0.82 | 16.51 | **6.89E-05** | 0.03 | 0.85 |
|  | 10 | (1,204) | 0.46 | 0.50 | 5.17 | 0.02 | 1.86 | 0.17 |
|  | 20 | (1,204) | 1.83 | 0.18 | 0.82 | 0.37 | 0.01 | 0.92 |
|  | 30 | (1,204) | 0.05 | 0.82 | 17.92 | **3.48E-05** | 0.22 | 0.64 |
|  | 40 | (1,204) | 1.40 | 0.24 | 81.97 | **<2.00E-16** | 0.89 | 0.35 |
| E_n_ | 5 | (1,204) | 0.03 | 0.86 | 53.73 | **5.28E-12** | 0.01 | 0.93 |
|  | 10 | (1,204) | 1.03 | 0.31 | 122.79 | **<2.00E-16** | 0.37 | 0.55 |
|  | 20 | (1,204) | 3.35 | 0.07 | 296.81 | **<2.00E-16** | 2.58 | 0.11 |
|  | 30 | (1,204) | 8.58 | **3.78E-03** | 7.99 | **<2.22E-16** | 7.99 | **5.12E-03** |
|  | 40 | (1,204) | 11.08 | **1.03E-03** | 367.90 | **<2.22E-16** | 17.53 | **4.20E-05** |
| BC_n_ | 5 | (1,204) | 8.88E-04 | 0.98 | 64.56 | **7.14E-14** | 0.32 | 0.57 |
|  | 10 | (1,204) | 3.02 | 0.08 | 147.71 | **<2.00E-16** | 0.46 | 0.50 |
|  | 20 | (1,204) | 4.28E-04 | 0.98 | 159.74 | **<2.00E-16** | 0.07 | 0.80 |
|  | 30 | (1,204) | 0.81 | 0.37 | 258.82 | **<2.00E-16** | 0.80 | 0.37 |
|  | 40 | (1,204) | 8.83 | **3.32E-03** | 350.44 | **<2.22E-16** | 15.36 | **1.21E-04** |

Note: P-values are uncorrected but those in bold are significant after a Bonferroni correction (p<0.05) correcting for 4 tests.

# **Table S9:** Summary of normalized global graph measures for weighted networks for the Oxford-Harvard atlas

| raw (partial) correlation as weight | simple denoising strategy | | |  |  |  |  |  | | complex denoising strategy | | | | | | |  |  |  |  |
| --- | --- | --- | --- | --- | --- | --- | --- | --- | --- | --- | --- | --- | --- | --- | --- | --- | --- | --- | --- | --- |
|  | correlations | |  |  | partial correlations | | | |  | | correlations | | |  | |  | partial correlations | | |  |
|  | values at time point 1 | | TRT (%) |  | values at time point 1 | | TRT (%) |  | | values at time point 1 | | | TRT (%) | |  | | values at time point 1 | | TRT (%) |  |
| **Normalized graph measure** | mean | SD | mean | SD | mean | SD | mean | SD | | mean | | SD | mean | | SD | | mean | SD | mean | SD |
| λ_n_ | 1.127 | 0.023 | 2.6 | 2.0 | 1.053 | 0.018 | 1.4 | 1.1 | | 1.128 | | 0.023 | 2.5 | | 1.9 | | 1.053 | 0.017 | 1.4 | 1.3 |
| C_n_ | 1.036 | 0.010 | 1.3 | 0.9 | 1.016 | 0.007 | 0.6 | 0.5 | | 1.036 | | 0.011 | 1.3 | | 0.9 | | 1.015 | 0.007 | 0.5 | 0.6 |
| E_n_ | 0.932 | 0.010 | 1.3 | 1.1 | 0.966 | 0.010 | 0.9 | 0.7 | | 0.930 | | 0.010 | 1.3 | | 1.1 | | 0.966 | 0.010 | 0.9 | 0.8 |
| BC_n_ | 0.906 | 0.032 | 4.4 | 3.5 | 0.980 | 0.023 | 2.5 | 2.0 | | 0.905 | | 0.032 | 4.1 | | 4.0 | | 0.980 | 0.021 | 2.2 | 1.8 |

| Main Effects |  | Denoising | | Correlation | | Interaction | |
| --- | --- | --- | --- | --- | --- | --- | --- |
|  |  | (S vs C) | | (Cor vs Parco) | |  | |
|  | df | F | *p* | F | *p* | F | *p* |
| λ_n_ | (1,204) | 0.25 | 0.62 | 37.26 | **5.13E-09** | 0.13 | 0.72 |
| C_n_ | (1,204) | 0.52 | 0.47 | 86.76 | **< 2.00E-16** | 0.71 | 0.40 |
| E_n_ | (1,204) | 0.19 | 0.66 | 11.25 | **9.51E-04** | 0.29 | 0.59 |
| BC_n_ | (1,204) | 1.61 | 0.21 | 30.40 | **1.06E-07** | 0.12 | 0.73 |

Note: P-values are uncorrected but those in bold are significant after a Bonferroni correction (p<0.05) correcting for 4 tests.

# **Table S10:** Summary of normalized nodal graph measures for weighted networks for the Oxford-Harvard atlas

|  | simple denoising strategy | | | |  |  |  |  |
| --- | --- | --- | --- | --- | --- | --- | --- | --- |
| A. raw (partial) correlation as weight | correlations | |  |  | partial correlations | | |  |
|  | values at time point 1 | | TRT (%) |  | values at time point 1 | | TRT (%) |  |
| **Normalized graph measure** | Mean  (range) | SD | Mean  (range) | SD | Mean  (range) | SD | Mean  (range) | SD  (range) |
| nodal average path length | 1.025 – 1.232 | 0.097 – 0.167 | 9.2 – 15.8 | 7.1 – 12.3 | 1.009 – 1.109 | 0.078 – 0.120 | 8.0 – 11.9 | 5.0 – 9.6 |
| local clustering coefficient | 1.018 – 1.056 | 0.024 – 0.042 | 2.3 – 4.2 | 1.7 – 3.3 | 0.999 – 1.034 | 0.033 – 0.047 | 3.1 – 5.4 | 2.7 – 4.1 |
| nodal efficiency | 0.816 – 1.388 | 0.297 – 0.440 | 30.2 – 50.5 | 21.6 – 36.8 | 0.915 – 1.378 | 0.302 – 0.496 | 30.9 – 45.0 | 22.1 – 32.2 |
| local betweenness centrality | 0.542 – 1.460 | 0.531 – 1.184 | 65.6 – 121.9 | 47.5 – 69.6 | 0.583 -1.355 | 0.501 – 1.050 | 68.4– 114.3 | 48.5 – 68.4 |

|  | complex denoising strategy | | | |  |  |  |  |
| --- | --- | --- | --- | --- | --- | --- | --- | --- |
| B. raw (partial) correlation as weight | correlations | |  |  | partial correlations | | |  |
|  | values at time point 1 | | TRT (%) |  | values at time point 1 | | TRT (%) |  |
| **Normalized graph measure** | Mean  (range) | SD | Mean  (range) | SD | Mean  (range) | SD | Mean  (range) | SD  (range) |
| nodal average path length | 1.041 – 1.231 | 0.096 – 0.171 | 9.1 – 15.0 | 7.2 – 11.4 | 1.006 – 1.108 | 0.072 – 0.120 | 8.3 – 12.2 | 6.4 – 9.3 |
| local clustering coefficient | 1.021 – 1.055 | 0.024 – 0.042 | 2.3 – 4.2 | 2.0 – 3.7 | 0.997 – 1.035 | 0.033 – 0.050 | 3.7– 5.3 | 2.5 – 4.2 |
| nodal efficiency | 0.818 – 1.377 | 0.282 – 0.433 | 29.0 – 46.7 | 21.2 – 36.6 | 0.945 – 1.336 | 0.322 – 0.472 | 31.5 – 43.8 | 22.5 – 31.5 |
| local betweenness centrality | 0.478 – 1.340 | 0.418 – 1.165 | 68.0 – 120.1 | 46.7 – 74.0 | 0.595 – 1.358 | 0.505 – 1.124 | 68.5 – 110.5 | 46.8 – 67.2 |

Note: the mean and SD represent the mean and SD of the nodal graph measure or TRT across subjects.

# **References**

Dimitriadis, S. I., Antonakakis, M., Simos, P. G., Fletcher, J., and Papanicolaou, A. (2017a). Data-driven topological filtering based on orthogonal minimal spanning trees: application to multi-group MEG resting-state connectivity. Brain Connect. 7, 661–670. doi: 10.1089/brain.2017.0512

Dimitriadis, S. I., Salis, C., Tarnanas, I., and Linden, D. (2017b). Topological filtering of dynamic functional brain networks unfolds informative chronnectomics: a novel data-driven thresholding scheme based on orthogonal minimal spanning trees (OMSTs). Front. Neuroinform. 11:28. doi: 10.3389/fninf.2017.00028
